# Supplementary material for: Integrated mental health for refugees: A realist theory building study
Source: PLOS Ment Health. 2026 Jan 30;3(1):e0000547. doi: 10.1371/journal.pmen.0000547 (PMC12857968; doi:10.1371/journal.pmen.0000547)
Supplement: S1 Text — (DOCX) [file pmen.0000547.s001.docx]

**S1 Text. Interview guide.**

Thank you for joining our deliberative dialogue session on realist evaluation. Before we begin, we want to acknowledge the “**We acknowledge and respect the lək̓ʷəŋən peoples on whose traditional territory the university stands and the Songhees, Esquimalt and W̱SÁNEĆ peoples whose historical relationships with the land continue to this day.”**

We wanted to review the purpose of the project and your voluntary participation. Would it be ok to audio record our session so that we can transcribe the dialogue for the purpose of analysis? (we will begin recording if there is agreement)

We anticipate that your time today will be approximately 1 hour. During this time, we will provide an overview of the project, realist evaluation and engage in interactive dialogue by using a Jamboard on the UVic secure Zoom. We will also provide a brief 10 min stretch refreshment break. We hope that by the end of our session we will have recorded an evidence-based summary of what you believe promotes integrated mental health care and services for refugees in BC.

My name is Nancy Clark and I will be facilitating the session. We have technical support provided by Drawing Change Consulting Inc. They will work with us to review the technical needs for different points in the meeting (small group work, different outside online tools) and assists attendees in navigating the online tools used during the session (eg slides, collaboration documents, chat, other tools as needed); Ensures reports and recordings are uploaded after the meeting.

As you know you have been invited to participate because you have knowledge and expertise in providing mental health support services for refugees as a primary care provider, settlement service provider or policy expert. The purpose of our convening and collaboration activities are to understand what promotes integrated mental health care for refugees in BC. Our objectives are to:

a) Build capacity for cross sector collaboration through a series of knowledge translation activities

b) Co-develop a working theory about what features of integrated care, i.e., practices, contexts

c) Co-develop a policy brief and knowledge translation summaries that will inform key knowledge users about what works, for whom and in what circumstance related to integrated refugee mental health

d) Use findings from our activities to inform our program theory and proposal for a larger operating grant. **(5 min)**

**Realist evaluation** (brief overview) To begin our session, we will review what is realist evaluation- realist evaluation is a theory driven approach to assess complex evidence such as policy, programmes, services and/or intervention. Many policies are implemented at local levels and in some cases these policies or programmes are not well understood. The purpose of realist evaluation is to better understand the programmes/intervention architecture so that it can be evaluated. Realist logic is not about does an intervention work, rather we are trying to find out how it works, for whom and under what contexts.

“**Context:** environment or backdrop of programmes

**Programme Mechanism**: how people respond to a programme’s resources

**Outcome:** The impact of the programmes resources activating in context”

In this project the intervention/programme is ‘integrated mental health care’ we want to know how it works to promote refugee mental health in BC. In other words what are the resources needed to provide integrated care? And how do people like you respond to the resource (socially and/or emotionally) the response is considered a mechanism in realist logic. For example: what is the mechanism thought to create integrated care?

To understand this, we first want to hear your thoughts about what you think integrated care is? How would you define it? (Use Jamboard to describe key concepts). Group works together to identify key concepts.

Before we start, we provide and example of a mechanism: “we are all housed under one roof which provides better integration”- describe what is it about that strategy that works? This helps us refine the program theory, i.e., integrated mental health care.

Group works together to brainstorm initial program theories. Concepts/ideas are recorded on the Jamboard. We will allow for approx. 15-20 minutes. When group decides we have enough concepts we will have a break. Following the break, we will return to wrap up. **(30 min)**

**10 min break**

Developing initial program theory sharing session

Group reflects on the different dimensions of programme theorizing that we learned and challenges in constructing IPTs. **(10 min)**

Wrap up:

Facilitator (NC) summarizes key CMO configurations, thanks everyone for their work and provides information on the World Café event**. (5 min)**
