# Supplementary material for: Integrated mental health for refugees: A realist theory building study
Source: PLOS Ment Health. 2026 Jan 30;3(1):e0000547. doi: 10.1371/journal.pmen.0000547 (PMC12857968; doi:10.1371/journal.pmen.0000547)
Supplement: S2 Text — (DOCX) [file pmen.0000547.s002.docx]

**S2 Text. Consensus-building exercise.**

**CONTEXTUAL CONDITIONS**

Alienation

**FIRING MECHANISMS**

Proactivity

Fragmentation

Connection

Burnout

Moral commitment

Stagnation

Trust

**Limited access and delays to access mental health care, unmet mental health needs, exclusion, resistance to care and fractured care delivery**

**Refugee client well-being, improved access to mental health care, client and service empowerment, ease of service navigation, retention and mental health recovery**

**OUTCOMES**

Integrated mental health care for refugees is improved when services are culturally and linguistically responsive, when system navigation is simplified and collaborative, when frontline providers are supported to deliver integrated, relational care, and when funding and policy environments incentivise long-term, holistic models rather than short-term crisis responses. Trust-building mechanisms, cultural alignment, and system-level collaboration drive client engagement, retention, and mental health recovery, while fragmentation, cultural mismatch, and workforce burnout inhibit access and continuity of care.

**SECTION 1 - Cultural and Relational Factors. How culture, language, stigma, and social frameworks shape access and engagement.**

- 1. Refugee clients are not all the same but collectively experience multiple barriers related to accessing mental health services and support navigating complex social, legal and emotional challenges tied to displacement and integration (C). When clients engage with specialized refugee mental health programs like those that offer community-based interventions (I) VAST refugee clients (A) feel supported when services reflect their entire context and needs (M). This leads to increased client empowerment, engagement and well-being, with benefits extending beyond the individual to families and communities (O).
  2. Clients who are navigating cultural stigma and resettlement (A) are embedded in communities where mental health stigma is prevalent, and privacy concerns are heightened (C). When services are delivered within or alongside close-knit cultural communities (I), and if providers share clients' cultural backgrounds (A), clients could fear that disclosing mental health concerns may lead to judgment or breach of confidentiality; hence, when confidentiality is assured, safety and trust triggered (M). This can then lead to openness and engagement with mental health care, with clients being more likely to disclose concerns (O).
  3. Refugee clients arrive in Canada from linguistically diverse backgrounds, accessing mental health services in unfamiliar settings (C). The provision of mental health care in the client's language (I) when clients and providers share a common language and can directly communicate (A) makes clients feel more comfortable, understood and safer when they can express authentically in language (M). This commonality enhances integrated mental health care's trust, engagement and treatment effectiveness (O).
  4. Clients from refugee backgrounds and cultural brokers who share similar characteristics (A) engage in a culturally complex environment, with language barriers and overwhelming service systems impacting the refugee's settlement (C). The involvement of the cultural broker who supports clients in navigating mental health services (I) makes clients feel more confident, less overwhelmed and better understood when someone can bridge cultural gaps and advocate on their behalf, at least initially (M). This improves service navigation, trust and culturally sensitive care (O).
  5. Clients whose cultural frameworks and expectations of care culturally differ from those assumed by the services (A) are engaging with mainstream mental health services, following a rigid and individualistic view of mental health care (C). Culturally uninformed care practices that do not reflect clients' lived experiences or cultural views (I) make clients feel misunderstood, disconnected or frustrated when care does not resonate with their values or ways of understanding distress (M). This can reduce therapeutic engagement, limiting the effectiveness of mental health support (O).
  6. Refugee clients (A) who arrive in Canada with diverse, complex and individual socio-political experiences (C) are met by services with reductive assumptions and generalisations about refugee experiences, often based on broad national or ethnic categories (I). This makes the client feel unseen, stereotyped or dismissed when assumptions are not grounded in their unique experiences, which triggers rejection (M). This can lead to frustration, disengagement and reduced trust in care (O).
  7. Refugee clients (A) are engaging with mental health services from diverse cultural backgrounds where mental health support is accessed through relational, collective and community-oriented approaches (C). When mental health services match this delivery model through collective practices (I), clients feel more at ease, respected and motivated to engage as cultural expectations and treatment received align (M). This can generate greater engagement and comfort with mental health services (O).
  8. Clients whose experiences, understanding or needs do not align with the dominant model of mental health care (A) are forced to navigate a system shaped by Western norms and assumptions about mental health, recovery and help seeing (I), all through a pre-existing unfamiliarity with the mental health system and its expectations (C). This triggers a feeling of alienation, confusion, and being overwhelmed by culturally unfamiliar plans and referral processes that act as a one-size-fits-all approach (M). This creates disengagement from services, as clients struggle to access or follow through with care, and their mental health needs are not met (O).
  9. Refugee clients (A) are resettling from their previous communities where mental health issues are heavily stigmatised, concealed or understood through non-Western frameworks (C). When Western mental health services provide culturally insensitive mental health service delivery (I), clients perceive the mental health services as pathologising, alien or misaligned, leading to fear of judgment or shame (M). This can lead to resistance or disengagement from care (O).
  10. In instances where refugee communities are stigmatized, they may mistrust formal mental health services (C). Refugee clients (A) who are introduced to culturally grounded peer support community initiatives aimed at promoting mental health awareness and engagement (I) feel more at ease, safe and curious about mental health when presented by trusted peers or community figures (M). This can lead to increased engagement with mental health services when approached through trusted, culturally resonant pathways (O).
  11. When refugee clients (A), whose cultural and linguistic backgrounds differ from dominant norms in the care provider host country (C), health and social care are less accessible services that provide culturally and linguistically accessible care (I), cultural and linguistic matching can trigger a sense of recognition, and trust, and relational safety (M). This linguistic support such as interpreter service can promote the development of stronger patient-provider relationships and facilitates meaningful engagement with services, ultimately leading to improved better outcomes (O).
  12. When refugee clients (A) access community health services with culturally rooted expectations of collective and community-based care (C) they encounter mental health services that rely on Western, individualistic models that treat everyone the same e.g. language ability (I), it can trigger feelings of alienation, cultural dissonance, and mistrust (M). This may disrupt communication, reduce perceived relevance of care, and ultimately lead to lack of culturally safe care and disengagement from services (O).

**SECTION 2 - System Navigation and Service Access. How people move through mental health and social services; problems with silos, complexity, fragmentation.**

**2.1** Care providers and refugee clients (A) interact through systems with multiple agencies involved in care delivery, lacking shared protocols or integrated workflows (C). This fragmented, uncoordinated process across mental health care (I) frustrates providers and clients as care stalls, not necessarily through shortages but due to inefficient communication and collaboration (M). This leads to stalled care, inconsistent waiting lists and delays in service access (O).

**2.2** Clients with mental health needs and frontline providers (A) navigate fragmented and disconnected intake pathways across services (C), with referral and transfer systems operating under siloed care and legal mandates, each with distinct processes, eligibility criteria and administrative requirements (I). As a result, clients feel overwhelmed, discouraged and excluded due to repeated burdensome administrative hurdles (M), which leads to the marginalisation of clients with mental health needs complex clients or exclusion from accessing care, often having to restart processes multiple times, which delays or prevents appropriate support (O).

**2.3** Clients attempting to access support and service providers working within narrowly defined mandates (A) are forced to navigate multiple services that lack coordination and shared responsibility (C), with the integrated mental health care delivery being fragmented through siloed mandates across sectors (I). These trigger stagnation as clients are bounced around services (M). This leads to stagnated care, with clients cycling between services, leading to unmet needs and disengagement from mental health services (O)

**2.4** In resource-limited and bureaucratically complex environments (C), a fragmented service system with rigid referral requirements (I) is implemented for clients relying on proactive advocates to navigate the system on their behalf (A). This leads to clients feeling supported (M) which can improve access to care (O)

**2.5** In complex cases where refugee clients need urgent and coordinated responses (C), service providers (A) attempt to work on the ground often through bottom-up advocacy-driven approaches, yet these clash with top-down service structures and bureaucratic mandates (I). This creates a sense of constriction, yet the provider maintains moral commitment (M). These delays hinder timely integrated mental health care (O).

**2.6** An often multi-often-multi-sector component treatment care cycle for refugees (C) requires service providers (A) to engage with multiple agencies to access different specialised care services (I). This leads to greater frustration and makes the system rely on individuals willing to persist through these challenges (O), which is often enabled by seasoned service providers who are familiar with the unpredictable ins and outs of the disjointed system referring based on trust, knowledge and trust expertise of the other providers (M).

**2.7** When service organisations (A) attempt to implement integrated care initiatives (I) within a context of fragmented and inconsistently managed government support (C), their ability to fully commit to integration is limited (O), because disjointed collaboration/communication institutional create confusion, delay, and misalignment across levels of the health and social care system (M).

**SECTION 3 - Organisational and Workforce Challenges. How agencies/providers operate internally — staffing, burnout, rigid workflows, reliance on champions.**

**3.1** Clients who experience ongoing or delayed-onset mental health challenges and providers constrained by refugee-label privileges (A) are forced to engage in system-imposed timelines and arbitrary definitions of functionality (C) within predefined classifications that determine service eligibility and duration (I). This can trigger a sense of unfairness and frustration as clients are being excluded based on arbitrary timelines or political labels rather than actual mental health needs (M). This creates a mismatch between service availability and actual client needs (O).

**3.2** Service providers and teams who are responsible for integrated mental health care (A) often collaborate with multi-agency teams working with refugee clients with varied mental health needs in a system where institutional targets drive service delivery (C), shaped by a market-driven, bureaucratic logic of performance metrics and outcome goals (I). These trigger pressure to meet institutional goals and performance indicators, leading to protective or territorial attitudes over clients to meet goals (M). This results in a breakdown in collaborative care, with teams struggling to share cases, confusing responsibilities, fragmentation of services and a negative impact on integration and client experience (O).

**3.3** Refugee clients with ongoing or complex mental health needs navigating discharge and recovery pathways (A) are forced to move through the system quickly due to a pressured environment and limited resources for long-term support (C), with rigid definitions of functionality and institutional processes and practices prioritising output, efficient and standardised metrics (I). This can lead to clients feeling unprepared or unsupported post-discharge, perceiving that their recovery needs are not fully acknowledged (M). This results in a premature discharge and a "revolving door" experience where clients cycle back into services shortly after leaving, with meets being unmet and adding pressure again to the system (O).

**3.4** Experienced, value-driven practitioners within the system (A) navigate fragmented systems with limited structural integration and formal coordination across services (C). The presence of a dedicated human agent in the service, which provides advocacy, moral commitment and willingness to work beyond formal role boundaries (I), leads to providers feeling ethically compelled to ensure clients do not fall through the cracks, drawing on personal values and professional judgement to make the best healthcare decisions (M). This leads to informal coordination of care, where service integration is sustained through the initiative of committed individuals rather than systematic support (O).

**3.5** Service providers committed to holistic care and clients engaging in deeper, trust-based conversations (A) interact through pre-existing challenges and mental health concerns, such as housing or legal status (C). A person-centred approach supported by service provider advocacy (I) makes clients feel seen, valued and understood when their broader needs are acknowledged, building trust and engagement (M). This leads to expanded and more effective care - mental health services begin to address interconnected needs (O).

**3.6** Service providers in mental health settings (A) work with refugee populations whose mental health needs are evolving, complex, and context-dependent (C). When rigid and outdated funding structures constrain how organisations and healthcare services are delivered (I), this causes provider frustration, feeling powerless, and diminished self-efficacy professional compromise when they are unable to respond to real-time needs (M). This leads to unmet client needs (O).

**3.7** When clients with unmet support needs (A) are being served in a mental health environment with clinician shortages and limited service capacity (C), the introduction of trained and supervised volunteers to provide non-clinical community-based mental health support (I) clients feel a sense of continuity and connection towards their care (M). This addition can improve access to mental health services and mental care engagement (O).

**3.8** Service providers and refugee clients (A) battle between managing the competing life demands of resettlement and unfamiliarity with the system (C), while providers invest time to build trust and rapport with refugee clients to engage in mental health care (I). Despite the efforts from providers, the competing life demands create a sense of devaluation for mental health for immediate logistical and family responsibilities (M), which could, in turn, create low attendance or engagement with mental health services (O).

**3.9** School personnel (A) may identify distress in refugee children, where schools become the primary point of contact for refugee children and their family's needs with limited mental health resources and a lack of apparent inter-agency collaboration (C). The referral between the school and the mental health system (I) is triggered by a sense of responsibility in the school to safeguard their children (M). This delays care for the children and their families (O).

**3.10** Services become fragmented and quality decreases (O) when a highly stressed workforce faces severe understaffing and workforce turnover (C) and when the delivery of mental health services are highly underfunded (I). This drives burnout, fatigue, and inconsistent patient engagement (M), thus driving service fragmentation (O)

**3.11** The lack of clear referral pathways resources being provided to the care ecosystem (I) coupled with a high need for specialised care and complex case profiles for refugees (C) leads service providers (A) to experience barriers to collaboration burnout and disengagement from their work (M) leading to cut corners, fatigue, and frustration between organisations results in refugees not getting the care they need-equity lens (O).

**SECTION 4 - Policy, Funding, and Governance Barriers. Bigger system drivers: funding, political will, structures that shape the whole system.**

4.1 Refugee clients (A) who present in settlement contexts are introduced to a system that prioritises acute presentations (C). These crisis-driven patterns within a system (I) mean the client must often reach a point of acute distress before receiving attention, which can cause reactivity (M). This delays access to long-term care and prevention as attention is placed on the moment of crisis (O).

4.2 Refugees’ (A) access to resources, including time, finances, and even energy, are influenced by their immigration status (C), leading to differences in access to care (I). This leads to fragmentation care (M), making it difficult for service providers to offer holistic are required to meet complexity of refugee mental health needs (O).

4.3 When government actors (A) operate in a context where funding decisions are shaped by shifting public attention and political interests (C), and where advocacy plays a key role in agenda-setting (I), funding is more likely to be allocated to issues that are highly visible and politically aligned (O), because self-advocacy and alignment with dominant narratives trigger responsiveness and perceived legitimacy within decision-making processes (M).

4.4 When policymakers (A) work within a context of decentralised governance and absent standardised reporting mechanisms (C), efforts to understand and address fragmented care are undermined (O), because the lack of coordination in reporting prevents the system from learning, adapting, or investing in integrative solutions (M).

4.5 When refugee clients and providers (A) operate in a context where shared language and cultural understanding are central to trust-building in care (C) but where there is limited availability of specialised linguistic providers (I) a persistent gap in service quality and accessibility emerges (O), because the unmet demand for culturally aligned care creates barriers to engagement and satisfaction (M).

4.6 When funders and health system leaders (A) adopt a strategic focus on prevention and mental health promotion (I) in settlement contexts (C), can lead to efficient, cost effective and client centered intervention (O), because providers are proactive (M).

Intervention

Context

Actor

Mechanism

Outcome

**SECTION 1 - Cultural and Relational Factors. How culture, language, stigma, and social frameworks shape access and engagement.**

1.1 Refugee clients are not all the same but collectively experience multiple barriers related to accessing mental health services and support navigating complex social, legal and emotional challenges tied to displacement and integration (C). When clients engage with specialized refugee mental health programs like those that offer community-based interventions (I) VAST refugee clients (A) feel supported when services reflect their entire context and needs (M). This leads to increased client empowerment, engagement and well-being, with benefits extending beyond the individual to families and communities (O).

1.2 Clients who are navigating cultural stigma and resettlement (A) are embedded in communities where mental health stigma is prevalent, and privacy concerns are heightened (C). When services are delivered within or alongside close-knit cultural communities (I), and if providers share clients' cultural backgrounds (A), clients could fear that disclosing mental health concerns may lead to judgment or breach of confidentiality; hence, when confidentiality is assured, safety and trust triggered (M). This can then lead to openness and engagement with mental health care, with clients being more likely to disclose concerns (O).

1.3 Refugee clients arrive in Canada from linguistically diverse backgrounds, accessing mental health services in unfamiliar settings (C). The provision of mental health care in the client's language (I) when clients and providers share a common language and can directly communicate (A) makes clients feel more comfortable, understood and safer when they can express authentically in language (M). This commonality enhances integrated mental health care's trust, engagement and treatment effectiveness (O).

1.4 Clients from refugee backgrounds and cultural brokers who share similar characteristics (A) engage in a culturally complex environment, with language barriers and overwhelming service systems impacting the refugee's settlement (C). The involvement of the cultural broker who supports clients in navigating mental health services (I) makes clients feel more confident, less overwhelmed and better understood when someone can bridge cultural gaps and advocate on their behalf, at least initially (M). This improves service navigation, trust and culturally sensitive care (O).

1.5 Clients whose cultural frameworks and expectations of care culturally differ from those assumed by the services (A) are engaging with mainstream mental health services, following a rigid and individualistic view of mental health care (C). Culturally uninformed care practices that do not reflect clients' lived experiences or cultural views (I) make clients feel misunderstood, disconnected or frustrated when care does not resonate with their values or ways of understanding distress (M). This can reduce therapeutic engagement, limiting the effectiveness of mental health support (O).

1.6 Refugee clients (A) who arrive in Canada with diverse, complex and individual socio-political experiences (C) are met by services with reductive assumptions and generalisations about refugee experiences, often based on broad national or ethnic categories (I). This makes the client feel unseen, stereotyped or dismissed when assumptions are not grounded in their unique experiences, which triggers rejection (M). This can lead to frustration, disengagement and reduced trust in care (O).

1.7 Care providers and refugee clients (A) interact through systems with multiple agencies involved in care delivery, lacking shared protocols or integrated workflows (C). This fragmented, uncoordinated process across mental health care (I) frustrates providers and clients as care stalls, not necessarily through shortages but due to inefficient communication and collaboration (M). This leads to stalled care, inconsistent waiting lists and delays in service access (O).

1.8 Clients who experience ongoing or delayed-onset mental health challenges and providers constrained by refugee-label privileges (A) are forced to engage in system-imposed timelines and arbitrary definitions of functionality (C) within predefined classifications that determine service eligibility and duration (I). This can trigger a sense of unfairness and frustration as clients are being excluded based on arbitrary timelines or political labels rather than actual mental health needs (M). This creates a mismatch between service availability and actual client needs (O).

1.9 Refugee clients (A) are engaging with mental health services from diverse cultural backgrounds where mental health support is accessed through relational, collective and community-oriented approaches (C). When mental health services match this delivery model through collective practices (I), clients feel more at ease, respected and motivated to engage as cultural expectations and treatment received align (M). This can generate greater engagement and comfort with mental health services (O).

1.10 Clients with mental health needs and frontline providers (A) navigate fragmented and disconnected intake pathways across services (C), with referral and transfer systems operating under siloed care and legal mandates, each with distinct processes, eligibility criteria and administrative requirements (I). As a result, clients feel overwhelmed, discouraged and excluded due to repeated burdensome administrative hurdles (M), which leads to the marginalisation of clients with mental health needs complex clients or exclusion from accessing care, often having to restart processes multiple times, which delays or prevents appropriate support (O).

1.11 Service providers and teams who are responsible for integrated mental health care (A) often collaborate with multi-agency teams working with refugee clients with varied mental health needs in a system where institutional targets drive service delivery (C), shaped by a market-driven, bureaucratic logic of performance metrics and outcome goals (I). These trigger pressure to meet institutional goals and performance indicators, leading to protective or territorial attitudes over clients to meet goals (M). This results in a breakdown in collaborative care, with teams struggling to share cases, confusing responsibilities, fragmentation of services and a negative impact on integration and client experience (O).

1.12 Refugee clients with ongoing or complex mental health needs navigating discharge and recovery pathways (A) are forced to move through the system quickly due to a pressured environment and limited resources for long-term support (C), with rigid definitions of functionality and institutional processes and practices prioritising output, efficient and standardised metrics (I). This can lead to clients feeling unprepared or unsupported post-discharge, perceiving that their recovery needs are not fully acknowledged (M). This results in a premature discharge and a "revolving door" experience where clients cycle back into services shortly after leaving, with meets being unmet and adding pressure again to the system (O).

**SECTION 2 - System Navigation and Service Access. How people move through mental health and social services; problems with silos, complexity, fragmentation.**

2.1 Clients attempting to access support and service providers working within narrowly defined mandates (A) are forced to navigate multiple services that lack coordination and shared responsibility (C), with the integrated mental health care delivery being fragmented through siloed mandates across sectors (I). These intrinsic flaws trigger a sense of feeling stuck and unsupported as clients are bounced around services that cannot or will not take responsibility (M). This leads to stagnated care, with clients cycling between services, leading to unmet needs and disengagement from mental health services (O)

2.2 Experienced, value-driven practitioners within the system (A) navigate fragmented systems with limited structural integration and formal coordination across services (C). The presence of a dedicated human agent in the service, which provides advocacy, moral commitment and willingness to work beyond formal role boundaries (I), leads to providers feeling ethically compelled to ensure clients do not fall through the cracks, drawing on personal values and professional judgement to make the best healthcare decisions (M). This leads to informal coordination of care, where service integration is sustained through the initiative of committed individuals rather than systematic support (O).

2.3 Clients attempting to access support and service providers working within narrowly defined mandates (A) are forced to navigate multiple services that lack coordination and shared responsibility (C), with the integrated mental health care delivery being fragmented through siloed mandates across sectors (I). These trigger stagnation as clients are bounced around services (M). This leads to stagnated care, with clients cycling between services, leading to unmet needs and disengagement from mental health services (O)

2.4 Service providers committed to holistic care and clients engaging in deeper, trust-based conversations (A) interact through pre-existing challenges and mental health concerns, such as housing or legal status (C). A person-centred approach supported by service provider advocacy (I) makes clients feel seen, valued and understood when their broader needs are acknowledged, building trust and engagement (M). This leads to expanded and more effective care - mental health services begin to address interconnected needs (O).

2.5 Service providers in mental health settings (A) work with refugee populations whose mental health needs are evolving, complex, and context-dependent (C). When rigid and outdated funding structures constrain how organisations and healthcare services are delivered (I), this causes provider frustration, feeling powerless, and diminished self-efficacy professional compromise when they are unable to respond to real-time needs (M). This leads to unmet client needs (O).

2.6 In resource-limited and bureaucratically complex environments (C), a fragmented service system with rigid referral requirements (I) is implemented for clients relying on proactive advocates to navigate the system on their behalf (A). This leads to clients feeling supported (M) which can improve access to care (O)

2.7 Refugee clients who present with urgent symptoms (A) are introduced to a system that prioritises acute presentations due to limited resources and high demand (C). These crisis-driven patterns within a system with limited capacity for long-term care (I) mean the primary crises are attended to, but the client must often reach a point of acute distress before receiving attention, which can cause distress and concern (M). This delays access to long-term care and prevention as attention is placed on the moment of crisis (O).

**SECTION 3 - Organisational and Workforce Challenges. How agencies/providers operate internally — staffing, burnout, rigid workflows, reliance on champions.**

3.1 When clients with unmet support needs (A) are being served in a mental health environment with clinician shortages and limited service capacity (C), the introduction of trained and supervised volunteers to provide non-clinical community-based mental health support (I) clients feel a sense of continuity and connection towards their care (M). This addition can improve access to mental health services and mental care engagement (O).

3.2 In complex cases where refugee clients need urgent and coordinated responses (C), service providers (A) attempt to work on the ground often through bottom-up advocacy-driven approaches, yet these clash with top-down service structures and bureaucratic mandates (I). This creates a sense of constriction, yet the provider maintains moral commitment (M). These delays hinder timely integrated mental health care (O).

3.3 Service providers and refugee clients (A) battle between managing the competing life demands of resettlement and unfamiliarity with the system (C), while providers invest time to build trust and rapport with refugee clients to engage in mental health care (I). Despite the efforts from providers, the competing life demands create a sense of devaluation for mental health for immediate logistical and family responsibilities (M), which could, in turn, create low attendance or engagement with mental health services (O).

3.4 School personnel (A) may identify distress in refugee children, where schools become the primary point of contact for refugee children and their family's needs with limited mental health resources and a lack of apparent inter-agency collaboration (C). The referral between the school and the mental health system (I) is triggered by a sense of responsibility in the school to safeguard their children (M). This delays care for the children and their families (O).

3.5 Refugee clients (A) are resettling from their previous communities where mental health issues are heavily stigmatised, concealed or understood through non-Western frameworks (C). When Western mental health services provide culturally insensitive mental health service delivery (I), clients perceive the mental health services as pathologising, alien or misaligned, leading to fear of judgment or shame (M). This can lead to resistance or disengagement from care (O).

3.6 In instances where refugee communities are stigmatized, they may mistrust formal mental health services (C). Refugee clients (A) who are introduced to culturally grounded peer support community initiatives aimed at promoting mental health awareness and engagement (I) feel more at ease, safe and curious about mental health when presented by trusted peers or community figures (M). This can lead to increased engagement with mental health services when approached through trusted, culturally resonant pathways (O).

3.7 When clients with unmet support needs (A) are being served in a mental health environment with clinician shortages and limited service capacity (C), the introduction of trained and supervised volunteers to provide non-clinical community-based mental health support (I) clients feel a sense of continuity and connection towards their care (M). This addition can improve access to mental health services and mental care engagement (O).

3.8 When refugee clients (A), whose cultural and linguistic backgrounds differ from dominant norms in the care provider host country (C), health and social care are less accessible services that provide culturally and linguistically accessible care (I), cultural and linguistic matching can trigger a sense of recognition, and trust, and relational safety (M). This linguistic support such as interpreter service can promote the development of stronger patient-provider relationships and facilitates meaningful engagement with services, ultimately leading to improved better outcomes (O).

3.9 When refugee clients (A) access community health services with culturally rooted expectations of collective and community-based care (C) they encounter mental health services that rely on Western, individualistic models that treat everyone the same e.g. language ability (I), it can trigger feelings of alienation, cultural dissonance, and mistrust (M). This may disrupt communication, reduce perceived relevance of care, and ultimately lead to lack of culturally safe care and disengagement from services (O).

3.10 Refugees’ (A) access to resources, including time, finances, and even energy, are influenced by their immigration path (C), leading to differences in what services they have available to access to despite their needs (I). This leads to an innate fragmentation between clientele (M), making it difficult for service providers to offer uniform services while limiting refugees’ access to care (O).

3.11 The lack of clear referral pathways resources being provided to the care ecosystem (I) coupled with a high need for specialised care and complex case profiles for refugees (C) leads service providers (A) to experience barriers to collaboration burnout and disengagement from their work (M) leading to cut corners, fatigue, and frustration between organisations results in refugees not getting the care they need-equity lens (O).

**SECTION 4 - Policy, Funding, and Governance Barriers. Bigger system drivers: funding, political will, structures that shape the whole system.**

4.1 Refugee clients (A) who present in settlement contexts are introduced to a system that prioritises acute presentations (C). These crisis-driven patterns within a system (I) mean the client must often reach a point of acute distress before receiving attention, which can cause reactivity (M). This delays access to long-term care and prevention as attention is placed on the moment of crisis (O).

4.2 Refugees’ (A) access to resources, including time, finances, and even energy, are influenced by their immigration status (C), leading to differences in access to care (I). This leads to fragmentation care (M), making it difficult for service providers to offer holistic are required to meet complexity of refugee mental health needs (O).

4.3 When government actors (A) operate in a context where funding decisions are shaped by shifting public attention and political interests (C), and where advocacy plays a key role in agenda-setting (I), funding is more likely to be allocated to issues that are highly visible and politically aligned (O), because self-advocacy and alignment with dominant narratives trigger responsiveness and perceived legitimacy within decision-making processes (M).

4.4 When policymakers (A) work within a context of decentralised governance and absent standardised reporting mechanisms (C), efforts to understand and address fragmented care are undermined (O), because the lack of coordination in reporting prevents the system from learning, adapting, or investing in integrative solutions (M).

4.5 When refugee clients and providers (A) operate in a context where shared language and cultural understanding are central to trust-building in care (C) but where there is limited availability of specialised linguistic providers (I) a persistent gap in service quality and accessibility emerges (O), because the unmet demand for culturally aligned care creates barriers to engagement and satisfaction (M).

4.6 When funders and health system leaders (A) adopt a strategic focus on prevention and mental health promotion (I) in settlement contexts (C), can lead to efficient, cost effective and client cantered intervention (O), because providers are proactive (M).
