# Supplementary material for: Integrated mental health for refugees: A realist theory building study
Source: PLOS Ment Health. 2026 Jan 30;3(1):e0000547. doi: 10.1371/journal.pmen.0000547 (PMC12857968; doi:10.1371/journal.pmen.0000547)
Supplement: S1 Table — (PDF) [file pmen.0000547.s003.pdf]

Settlement service organization					
**Experiential**	**Quote**	**Inferential**	**Dispositional**	**Consensus**	**Final**
Financial organisational limitations lead to inflexible systems and interventions that cannot respond to complex and dynamic needs of this population, leaving those caring for clients without options (sense of helplessness)	*Like get funded. You're like, OK, this is not working, but we have 3 or 4 years of this, but it's like, why the? It's in. It's everywhere, right? Even in our contracts like there's no ability to react within the dollars that you're given, sometimes to change things as you learn.*	Specific and limited funding prevents organisations from adapting to dynamic/complex refugee health care needs. social determinants of health that require expanded collaboration and integrated support<br>and fulfilling a possibly outdated goal stipulated by the original funding contract	This is shaped by rigid expectations embedded in funding contracts, where predefined goals set during negotiations remain fixed, even when program needs evolve over time. A broader theory suggests that limited funding for refugee mental health stems not only from resource constraints but also from political values that frame immigrants as a burden on public systems, ultimately resulting in fewer resources allocated to support their mental health and wellbeing.	Rigid and insufficient funding structures restrict service providers' ability to adapt care to evolving client needs, leaving both clients and providers constrained by outdated or impractical funding agreements.	Inflexible funding models and limited financial resources create significant barriers to responsive, client-centred care. As client needs shift and grow more complex, service providers are left without the tools to adapt, leading to frustration, unmet needs, and a system that struggles to deliver on its intended outcomes. This rigidity increases the risk of refugees falling through the cracks, contributing to negative health outcomes and deepening existing inequities.
Many participants in this dialogue suggested that funding constraints and payment requirements create obstacles for service users.	*"The second part I think for many families is the fees you know, am I going to pay for this? Because again, by the time they came to me, it's been maybe a while, whether I'm meeting people who are existing ICA client, but because this is my first year or they've went through this one way or another, and at that time, you know, issues happen at school and we're starting to get referrals, it they don't have this, you know, funding that they have. And so we really need an affordable place."*	It is plausible to claim that current resource allocation is inequitable across services (example some schools may have settlement workers in schools where they are more culturally aware of refugee needs)<br>This creates an inefficient system toward mental health care is insufficient to create a seamless, integrated mental health network, leaving service users struggling to access affordable care.	The inferred phenomenon is driven by decision-makers' failure to prioritise integrated mental health care, resulting in a growing disconnect between what service providers strive to deliver and what is actually feasible within the confines of financial constraints and funding limitations. True integration must extend across sectors—including schools, where mental health challenges often first become visible—and requires dedicated	See above	See above

			processes and resources to ensure effective linkage and coordination between these settings.		
In resource limited care settings, cross-sector requests for care of refugees requires greater willingness for other organisations to accept care, meaning patient advocate champions working in different parts of the system are critical	*"So they, exactly. So they do the medical screening and [redacted], we agreed with the doctor there even if they don't need it he's referring everyone and he said, "I want to do this for everyone." Yeah and I think you're getting lots of referrals because of that."*	In a fractured service landscape, patient advocacy becomes increasingly important, as navigating access often hinges on obtaining appropriate referrals. Embedding values of equity within the system supports advocacy efforts by ensuring that referrals are used not as gatekeeping tools, but as mechanisms to promote fair and timely access to care.	This occurs when rigid boundaries between organisations mean that each service requires a formal referral for access, placing additional pressure on the efficiency and coordination of care entry points. However, when advocates or champions within the system are known to be receptive to refugee referrals, the likelihood of successful referral increases, thereby improving access to care and mitigating some of the system's structural barriers.	Recognising that in under-resourced and fragmented systems, effective access to mental health care for refugees often hinges on proactive referral pathways and the presence of strong values of equity and patient advocates across sectors.	Systemic gaps and limited resources make coordinated care difficult to deliver without the intervention of dedicated advocates who can navigate rigid referral requirements. These individuals act as crucial bridges, helping to ensure clients don't fall through the cracks in a system where the will to provide care often exceeds the means to do so.
Many participants in this dialogue strongly suggested that lack of availability of resources exists, which impacts the way they can provide mental health support	*"And one time we received a phone call from Ottawa about a Syrian family that was arriving, and they knew that they would be needing some serious mental health, and they asked do we have the resources to welcome that family here. Because many other places would not have the resources."*	It is plausible to claim that the gap between what staff intend to provide and the resources available creates a disconnect between planning and execution, ultimately impacting the quality of care delivered. This disconnect becomes particularly critical when accepting new refugees, as doing so requires adequate resources to support them; if those resources are lacking, refugees may be resettled elsewhere—often in a different province or city—highlighting the systemic limitations in meeting demand.	The inferred phenomenon—namely, the integration of services and mental health care—is dependent upon broken and outdated systems that widen the gap between intention and execution. As a result, the workforce is constrained by systemic limitations, often leading to frustration and a sense of helplessness in delivering adequate support. However, when staff possess strong knowledge of available resources and have		See above

			experience navigating the system, their capacity to support refugee mental health is significantly enhanced, helping to bridge some of these structural gaps.		
Participants described how clients with urgent needs tend to reach out to the system, showing a greater willingness to engage with different points of care, such as settlement services or schools. This pattern reflects a broader trend in which individuals are more likely to seek help during times of crisis, often because their immediate focus is on meeting basic needs such as housing, employment, and education—needs that must be addressed before mental health concerns can be fully acknowledged or managed.	*I'm seeing a very clear sign of them saying "I want to, you know, like I want to end my life" something like this otherwise they really didn't see a way to be involved. So I called the police to have a wellness check on her, and they ended up sending her to the hospital that day because they really felt like this is, you know, not safe for her or the children. And she spent, I think the rest of the week and the weekend and then got out with then a social worker being assigned, you know, having a meeting with her regularly, a doctor to see and so on.*	Patients with more urgent needs may be more willing to engage with the care system, creating opportunities to identify not only their own mental health needs but also those of their wider family. However, refugees often delay seeking help from the formal mental health system until they are in acute crisis, a pattern influenced by multiple factors—including access barriers, stigma, and cultural expectations. This dynamic is further shaped by gender-specific responses and the availability (or lack) of gender-sensitive resources, which can significantly affect when and how individuals seek support.	Effective care is dependent on the system's active vigilance and consistency, with providers prepared to respond promptly when cases arise. At the same time, accessing care relies heavily on trust, culturally safe support, and clear knowledge about who to approach for help. However, the absence of adequate prevention and mental health promotion resources for refugees further limits early engagement, leaving many needs unmet until they escalate into crises.	While clients with urgent needs may trigger quicker engagement with the system, the overall effectiveness of care still depends on the system's capacity to respond—something often limited by strained resources and inefficient referral processes.	Tension between client readiness and system responsiveness: even when clients are willing to engage—often in moments of crisis—the system's limited capacity and overstretched referral pathways can hinder timely and effective care. This mismatch risks both provider burnout and missed opportunities for early intervention, especially for families with broader, interconnected needs.
Many participants in this dialogue strongly showed their concerns about whether services have the capacity to handle referrals effectively, and whether the referral system is useful in resource-limited contexts.	*"We get a lot of referrals, the difficulty because there's always some issues that you only serve God. And we get refugees and immigrants from everywhere."*	It is plausible to claim that the existing pressures within healthcare services increase the likelihood of burnout and disengagement as capacity and resource are mismatched for the referral system to be efficient, ultimately leading to negative outcomes for both providers and service users.	The inferred phenomenon is dependent upon the presence of a resource-strained and high-stress healthcare environment, where chronic understaffing and systemic instability generate frustration, helplessness, and disengagement among all stakeholders.		See above

Participants debated the role of volunteers stepping into positions of care without professional training, and the needs of the system for experienced and supervised volunteers.	*"...the program you're talking about, I did that overseas. In fact, this was modelled after the groups I recruited working with Doctors Without Borders in every country and they were closely supervised, but they were able to fan out and they were able to meet people because many of these countries there were no counsellors, clinical counsellors. One psychiatrist for the whole country in some places. And the work they've been able to do was absolutely amazing."*	Volunteers may bridge service gaps by providing non-clinical community support in resource limited settings	This occurs when there are not enough clinicians available to serve a given community, and in many cases, the population in need may benefit more from basic peer support than from formal clinical assessment. To bridge this gap, volunteers play a vital role in addressing the social care needs of refugees—such as system navigation and peer support—which can significantly contribute to promoting mental health and overall wellbeing.	In the face of clinician shortages and overstretched systems, trained and supervised volunteers can play a critical role in extending support, especially for non-clinical and community-based needs.	Urgent need for creative, integrated solutions in under-resourced systems. While professional care remains essential, supervised volunteer involvement can help bridge service gaps, relieve pressure on limited clinical staff, and provide meaningful, community-grounded support—especially when designed with clear structure and oversight.
Many participants in this dialogue strongly suggested that issues related to service integration, workforce shortages, and the burden placed on fewer people taking on multiple roles exist, which impact on the quality of care being provided.	*"There needs to be a referral from a medical doctor and that was difficult right from the start. Then we only have two of us who were able to register with [redacted] and I am not taking too many clients anymore. And so the other one is in [redacted] and she was overloaded with new refugee clients. So then [redacted] becomes useless because we can't refer anymore once she's full."*	It is plausible to claim that service providers are seeking more integrated systems and closer relationships with other providers to facilitate seamless care and better service coordination.	The inferred phenomenon is dependent upon growing frustration and exhaustion among service providers, who frequently share examples of how the system is failing both themselves and the refugees they serve. While integrated mental health care relies on providers' knowledge of how to navigate medical insurance systems to access specialised services, such knowledge becomes ineffective when those medical services are already overwhelmed, rendering access to care inconsistent and often unattainable.	See above	See above

Participants discussed how family stress plays a role in how they manage cases, however with an already resource restricted system, care procedures stagnate in the absence of adequate care	*And with domestic violence we've got a lot of families with domestic violence. It's a lot of work, especially if the woman decides I don't want to have this anymore. And if she wants to leave with the children then you have to find the transition house where she's understood having to settle her in and then the man also needs help...*	Cases involving families and family tension are often more resource-intensive and can present with greater urgency, which may lead to treatment delays and increased workloads for providers. These complexities are further compounded by the need for gender-specific care, particularly in situations involving domestic violence, where tailored interventions are essential to ensure safety and provide appropriate support for all family members.	This is dependent on pre-existing resource limitations that providers face when attempting to support families, within the context of an already strained workforce responsible for attending to multiple complex cases. These challenges are exacerbated by the lack of established gender-specific support systems, making it even more difficult to respond effectively to the distinct needs that arise within family dynamics, particularly in sensitive contexts such as domestic violence.	Complex family cases, particularly those involving violence or crisis, require urgent and intensive support; however, systemic bureaucracy and limited resources often delay timely and appropriate intervention. Addressing these situations effectively requires acknowledging the root causes of domestic violence, which are often linked to family stress, and ensuring that enhanced resources are available—ones that are both gender-sensitive and culturally safe.	Critical gap between the complexity of frontline care and the inflexibility of the system tasked with delivering it. When structural barriers imposed from top-down and limited resources intersect with high-need family cases, providers face overwhelming challenges to deliver integrated care bottom-up that delay care and compromise support, reinforcing the urgent need for more responsive and adequately resourced mental health and psychosocial services
Many participants in this dialogue strongly suggested that structural challenges and bureaucratic barriers prevent efficient service delivery	*"Yeah yeah it's a red tape issue. It's politics."*	It is plausible to claim that bureaucratic and structural barriers prevent mental health care from being implemented in ways that reflect the realities of frontline experiences and needs, resulting in a persistent disconnect between policy and practice. These challenges are further compounded by systemic issues such as budget cuts to immigration-related funding, which have a cascading effect on the services and supports intended to promote refugee integration and wellbeing.	The inferred phenomenon is shaped by legal and moral restrictions imposed through top-down decision-making, while service delivery typically operates from the bottom up. This misalignment between structural directives and on-the-ground implementation leads to inefficiencies and significant challenges in delivering effective care. At the heart of this issue is the broader problem of inequitable health care,	See above	See above<br><br>"As many studies show, Canadian society is not free of discrimination based on gender, racialization, class, sexual orientation, language, disabilities, etc. Programs directed at the least privileged in our society offer essential support to communities seeking equity. If such programs are cut, how does the federal government intend to tackle the equity challenges that people face from coast to

			where systemic disparities further hinder access and outcomes for marginalised populations, including refugees.		coast"? Canadian Council for Refugees 2025
Participants discussed the role of patients' perceived priorities, stemming from a difference in understanding about the care system and busy schedule, care becomes deprioritised even when needed.	*"And I would say the last point of that is also fitting the support within their busy schedule. So when I'm talking about my bag, the majority of my clients who have an average of four or more kids and most of the time it's not one kid who gas the issues it's more than one. Fitting scheduling those kids in the places and if the mother doesn't know how to drive and they're only depending on the father, maybe there's no father, you know, whatever the situation is just that's why I think many of them also stop because like I can't handle, you know, going and bringing and scheduling and finding, you know that that timing"*	It is suggested that existing understanding and the amount of resources that a refugee has access to greatly dictates their ability to make time for mental health care	This occurs when mental health care systems are unable to meet refugees where they are—for example, through home visits or streamlined, accessible services—resulting in fragmented care spread across multiple appointments and organisations. This disjointed approach is further complicated by dominant biomedical frameworks that define mental health primarily in terms of symptoms and crisis, often neglecting the social dimensions of wellbeing, including health promotion, prevention, and the broader context of resettlement.	While meaningful, relationship-based care is essential for effective mental health support, refugees' competing priorities, limited resources, and structural barriers often prevent sustained engagement with services.	Tension between the need for consistent, trust-based care and the realities of refugee clients' daily lives—marked by logistical, familial, and structural challenges. Without flexible, accessible service delivery models that meet clients where they are, even well-intentioned and relational care risks being deprioritised or abandoned altogether.
Many participants in this dialogue suggested that it is important to spend time with clients to understand their needs and symptoms to provide the best quality of care possible.	*"But honestly, like the kind of support in an afterwards becomes more like social worker. Like OK, let's do this and then you know it's off. That is a kind of a person who needs someone on more regular checkups more, you know."*	It is plausible to claim that quality mental health care is built on strong relationships with refugees, where meaningful social connections and a focus on social care serve as a foundational pillar of successful care and recovery.	The inferred phenomenon is dependent upon the presence of trust and rapport-building, particularly for individuals with pre-existing trauma histories. Establishing a strong therapeutic relationship is essential to promoting engagement and alignment with their care plan.	See above	See above

Participants described the central role of children in a system that does not serve them: namely, their behaviour at school indicating family stress	*this is how I'm I get involved mostly with the cases and when do we need to refer the kids? It's because the kids are already showed, you know, certain behaviour or concerns at school. The counsellor have seen them. Things have gone to a point where the school counsellor cannot deal with how they can support that child anymore. So they ask for our help for a referral.*	Children with complex cases surpass the services that school provides them, leading them into the overworked settlement system where child-specific services are sparse	This occurs when schools draw firm boundaries between their educational responsibilities and those of mental health professionals, often resulting in a reliance on the mental health care system to take over complex cases. However, when schools are equipped with resources such as settlement workers, there is a greater likelihood that children's mental health needs are identified and supported early, fostering more integrated and responsive care within the educational environment.	While schools often act as the first point of identification for mental health concerns in children, they are not adequately equipped or resourced to manage complex trauma cases, resulting in over-reliance on an already overstretched settlement and mental health system.	Children often become the entry point into a fragmented care system, with schools bearing the responsibility of flagging concerns but lacking the capacity to respond effectively. Without stronger coordination between education, settlement, and mental health services, vulnerable children risk being passed between systems that are neither fully prepared nor sufficiently integrated to meet their multifaceted needs.
Heavy reliance on school as a point of care (? or intake?) leads to relying on personnel who do not necessarily know how to manage a case. Participants discussed the importance of communication between settlement, MH support, and school agencies to better address migrant needs.	*Fitting scheduling those kids in the places and if the mother doesn't know how to drive and they're only depending on the father, maybe there's no father, you know, whatever the situation is just that's why I think many of them also stop because like I can't handle, you know, going and bringing and scheduling and finding, you know that that timing. So when the school offers, you know, the counselling they say do whatever you want, but if it's at school. But going back to counselling at school, either they are overwhelmed with the cases they have in their own school or they say we're*	Positioning schools as points of care for mental health can be risky, as they are not always equipped to manage complex trauma cases or allocate resources beyond their educational mandate. While education systems often serve as a first point of contact for mental health interventions, they are frequently under-resourced to provide the specialised support needed by refugee families, highlighting the need for stronger cross-sector collaboration and investment.	This happens when the broader system struggles to identify clients—particularly families—due to its distance from everyday family spaces, while schools naturally fill this role because of their proximity to children and their caregivers.	See above	See above

	not equipped enough for handling the trauma and the issues				
Many participants in this dialogue suggested the effect of cultural stigma and previous experiences on willingness to engage with mental health services	*"Ukrainians, there was such a strong stigma against mental health. They want to look really strong and they are strong and resilient, but underneath it they really having a very hard time, a lot of homesickness, lot of grieving, but you don't know that unless you get to see them several times and see how they are doing and what's happening."* *t*	It is plausible to claim that cultural differences and stigma in mental health perceptions shape how support is initially received, with some individuals interpreting interventions as either helpful or, conversely, as unnecessary or even insulting.	The inferred phenomenon is dependent upon deeply rooted cultural preconceptions of mental health that may not align with the formal service provision model. This disconnect can alienate refugees from accessing care, as mental health services may be perceived as treating a "problem" they do not recognise as such.	Cultural stigma and differing worldviews around mental health can create a disconnect between refugee clients and Western mental health systems, leading to hesitation or refusal to engage with available services. Stigma is a barrier to mental health care globally for people of forced migration they experience other forms of discrimination so may need to look strong	Western, individualistic models of care may clash with refugees' cultural values, especially when mental health is stigmatised or traditionally addressed through community-based support. Without culturally responsive approaches that honour these differences, formal services risk being perceived as irrelevant or even threatening, leading to disengagement and unaddressed mental health needs.
Participants in this dialogue suggested that imposing Western models of care can alienate refugees who come from community-based support systems.	*"Yeah, I just, I want to just on that like the only like you know, it's always like the Western approach. We don't see that you know that community-based approach from having that. So that's the issue. That you know that having in Canada, right?"*	It is plausible to claim that Western, individualistic approaches to mental health care may not align with the expectations and cultural understandings of refugees, leading to disengagement and mistrust of services	The inferred phenomenon is dependent upon the existence of influence of cultural norms and differing perceptions of mental health, where discussing mental health openly may be culturally inappropriate or misunderstood, creating a barrier to engagement with care.	See above	See above
Participants in this dialogue highlighted the importance of cultural ambassadors, peer support, and community-led initiatives in improving engagement with mental health care. Cultural ambassadors, often operating as volunteers or non-regulated community workers, serve as	*"And speaking of language in that sense, not just the language, but like seeing where their core is. So, let's say if a Christian devoted, you know, a person who goes to church no matter where they are in, you know the universe. And you start from the conversation from the*	It is plausible to claim that cultural ambassadors and peer support networks act as crucial bridges between services and service users, providing reassurance that accessing services is safe and promoting trust between providers and communities.	The inferred phenomenon is dependent upon the existence of human connection and empathy in understanding trauma and hardship. When people feel seen and supported within familiar cultural and social	Community-led, culturally anchored approaches—such as involving cultural ambassadors and peer support—are key to improving engagement, especially when mental health services are grounded	Culturally embedded, grassroots approaches promote trust and participation far more effectively than top-down, imposed models. By meeting people where they are—socially, spiritually, and culturally—community-led initiatives help reduce

valuable resources in promoting integrated mental health care for refugees by bridging cultural gaps and promoting trust within communities.	*church. You know, you have guest speakers at the church or you ask, you know, like for this, you know, I know they have like, lunches and kind of, you know, social events, but you get, you know, someone like the volunteers of the ambassadors, you know, talking there."*		spaces, they are more likely to engage with services and integrate into the community.	in familiar, trusted spaces.	stigma, promote belonging, and create accessible, meaningful pathways into mental health care for refugee communities.
Participants in the dialogue argued whether mental health services should be designed in a way that responds to community needs or are imposed from above	*"And we got a grant of $25,000 for this particular program. But we started the program way before we got the grant. So we always start programs before we know how it's going to be paid."*	It is plausible to claim that community-based mental health approaches align more closely with refugees' pre-existing cultural expectations of care, thereby increasing their engagement and willingness to access services. However, despite their potential, such programs often lack dedicated funding, with support contingent upon first demonstrating effectiveness—highlighting a systemic barrier where promising initiatives must prove their value before receiving the resources needed to succeed.	The inferred phenomenon is dependent upon the presence of cultural norms that emphasise collective support and social connection, where individuals feel more comfortable and engaged in care when it is provided in a familiar, community-oriented setting rather than through Western individualistic models.	See above	See above

**Mental Health organization**					
**Experiential**	**Quote**	**Inferential**	**Dispositional**	**Consensus**	**Final**
The transfer/referral system marginalises clients who have more complex needs. The disconnect between all the service components adds	*The most challenging part for the line of social determinants, where the client has no documentation and we will have to literally start from zero. First of all,*	Clients with more complex needs may expose them to the challenges posed by a fractured system due to the multiple processes they need to navigate	This is dependent on the existence of a system that operates on different care mandates and are siloed in their operation, leading to the existence of multiple	Highlight both the problem (siloed systems with multiple processes) and the desired or inferred preference (streamlined systems for better care)	While the current system operates through siloed care mandates, resulting in fragmented and duplicative processes for clients, there is a structural preference—

several intake processes, eligibility requirements and “hoops” to jump over essentially that the process is even more burdensome for them than receiving treatment	*you know we need some BC ID. Where do we get that from? OK, call the ID bank and we produce a document that's used [in] our services as a reference for them to get that ID. And once they get a BC ID then other services will be opened up to them. So, all of that involved the case management follow up the. The constant follow up because some clients will be missing like, 'I can’t this is just too much for me to handle.*		processes and programmes that clients need to go through		both implicit and explicit—for more streamlined workflows that would allow providers to concentrate on care delivery and clients to receive high quality care rather than navigate administrative inefficiencies
**1.3.** The participant in this dialogue described the role of structured referral pathways and integration between teams in ensuring continuity and reducing confusion.	*“The Paris system is, is set up to, so a good example is in the Paris system we have the what we call the rehab team and the rehab team is comprised of the OT, the break therapist, myself, the employment counsellor and, who is on that team, the yeah, that's just, that's just the four of us on the rehab team. So, we have our own referral system. So, anything that comes through that system this, that will be our immediate focus.”*	1.3 It is plausible to claim that a structured referral system facilitates team integration, improving coordination and ensuring that cases are managed efficiently without unnecessary confusion or duplication of effort. This is consistent with findings from the narrative review by Uribe et al. (2023), which identified data sharing as a key component in the successful integration of health and social care services.	1.3 The inferred phenomenon is dependent upon the existence of a structural preference for streamlined processes, which enables providers to focus on care delivery without being distracted by administrative burdens or inefficient workflows.	See above.	See above.
Together but separate: while the system is set up to operate as one unit in their own respective corners, having different mandates leads to different evaluation metrics, leading to different outcome goals. This then leads to competition and a	*"We are supposed to be an integrated team that more often than not that's not the case. For instance, I work between two teams. I work between the substance use and the mental health team, and these teams have completely different mandates. But one client*	Diverse care mandates may lead to stronger boundaries between organisations, making it difficult to share cases across organisations	This is dependent on organisations having specific and distinct organisational goals and even funding sources, which in turn further shapes their attachment to their mandates and their hesitance to adopting new	Acknowledging that while teams are intended to work collaboratively within an integrated system, differing organisational mandates, metrics, and goals contribute to fragmentation and territorial behaviours, ultimately undermining collaborative care.	Systemic fragmentation arises not from interpersonal issues but from structural and organisational divides shaped by distinct mandates, performance pressures, and funding structures. This promotes competition rather than collaboration, making integrated care challenging despite shared intentions.

territorial attitude over clients across the system.	*can have both conditions. They do have mental health issues, but they also have substance use issues. So, then the question is who client is this? Is this client more for mental health related focus or is it more for substance use counselling kind of focus? And basically it's a competition between the teams because we are supposed to be integrated, but we have separate mandate in terms of who gets what services."*		practices geared to integration		
**1.6.** The participant in this dialogue strongly suggested that different teams sometimes compete for clients, prioritising organisational goals over collaborative care.	*"And basically it's a competition between the teams because we are supposed to be integrated, but we have separate mandate in terms of who gets what services".*	1.6 It is plausible to claim that competition between teams creates tensions that hinder the seamless integration of mental health care, leading to fragmented service provision.	1.6 The inferred phenomenon is dependent upon the influence of a market-driven, bureaucratic logic in healthcare, where service provision is shaped by meeting institutional targets and performance metrics rather than promoting holistic, collaborative care.		See above.
Client needs are not being sustainably met after they reach care "success", defined in this system as "functionality", leading to a revolving door of care, discharge, mental health challenges and a never ending stream of clients.	*"The, the argument is that well we have too many other clients that are coming in that are being filtered in, so if we don't, if we don't clear the list then we are going to be all overworked and, and, and, and we are under staffed [...] So, you have to discharge, and the goal is to get other people [...] But the question then becomes why if we send somebody to the back door, they always*	Institutional processes that systematise care often conflict with the realities of mental health support, resulting in client needs being consistently unmet. Time-limited care models are especially problematic, as mental health care is fundamentally relational and depends on sustained, supportive connections. When these relationships are prematurely terminated, individuals are left with reduced support and	This is dependent on the rigid attachment of an institution to process and procedures that may favour systemisation at the cost of personalised care	Recognising that systemic pressures to discharge clients quickly—driven by institutional efficiency and capacity constraints—conflict with the ongoing, often non-linear nature of mental health recovery, resulting in a cycle of repeated service use.	A system that prioritises throughput over sustained recovery, where discharges are more reflective of institutional demands than client readiness. This creates a revolving door effect, where individuals re-enter care due to unmet long-term needs, highlighting a misalignment between system processes and the realities of mental health support.

	come [back] to the front door? So, what is the goal we are hoping to achieve? Send them to the backdoor because they have housing and then couple months after they come through the front door like 'Oh, hi. Oh, you're back.'"	fragile systems that should instead be long-term and adaptable.			
The participant in this dialogue strongly suggested that a tension exists between discharging clients quickly due to system constraints and the high likelihood of relapse when care is not sustained.	*"To most of the people, most of the people we "discharge" in the quotation often are back in the system within couple of months or weeks. And then the question is it even necessary to discharge?"*	It is plausible to claim that premature discharge leads to unmet needs, increasing the likelihood of relapse and repeated referrals, which ultimately contributes to service congestion.	The inferred phenomenon is dependent upon systemic pressure caused by high service demand and limited staff capacity, where discharges may serve institutional efficiency but fail to align with clients' mental health trajectories, reinforcing a cycle of re-entry into care.		See above.
In a system that aims to be integrated, but in practice is siloed, the care/referral process contractics one another leading to stagnated care / client experiencing long lasting limbo in the system.	*"So, if we send a client to Duncan House, they are sent back to us because they have mental health issues. We send them to them for housing, they send them back to us. So the client is kind of flipping between these two services."*	The referral process may become counterproductive when organisations fail to work in unison, leading to fragmented care pathways. As a result, clients with addiction issues may receive support, but those with co-occurring or compounded disorders—including mental health conditions—often fall through the cracks due to the lack of coordinated service delivery.	This occurs when organisations are unprepared or unwilling to collaborate on case management and maintain inflexible intake procedures, resulting in cyclical or inefficient referral patterns. The persistent separation of mental health and addiction as distinct, unconnected issues further complicates efforts to integrate services, ultimately hindering comprehensive and coordinated care.	Recognising that in a fragmented and siloed system, rigid eligibility criteria and contradictory referral practices prevent services from responding flexibly to client needs, resulting in stagnated care and systemic inefficiencies. At the core of this issue is the separation of mandates between mental health and addiction services, which are often viewed and operated as distinct systems—undermining efforts to provide cohesive, integrated care.	Bureaucratic processes and a lack of inter-organisational collaboration leave clients in limbo, bouncing between services without their needs being met. This reflects a deeper structural issue where procedural rigidity and siloed mandates override holistic, client-centred care.
The participant in this dialogue strongly suggested that a gap exists between the systems used to access care and the realities of client	*"It's ,it's really mind boggling for me. I have to admit. Client's rejected by AAC because they're not eligible, but they have all these needs. They have*	It is plausible to claim that eligibility criteria often take precedence over actual client needs in refugee mental health care, creating systemic barriers to support.	The inferred phenomenon is dependent upon a risk-averse, bureaucratic system where rigid eligibility standards are enforced to minimise	See above.	See above.

experiences, leading to inefficiencies or unmet needs.	*housing issues. They have, um, they have a visible mental, mental health challenges, but because they are not eligible for the service provide if you rejected and so they do attend our mic drop-in groups that I conduct because it's a drop-in."*		liability and resource allocation, even at the cost of addressing pressing client needs.		
Integration relies on the diversity and skillset of the team because the client needs are often complex and overlapped. The process is broken down into bits to treat individual aspects of a complicated problem, but in practice this work can't be done in silos. Without a skilled workforce, that complexity gets flattened.	*"We are not necessarily concerned about the other aspect of the client. We are just concerned about that piece. [B]ut that client again is going to distribute it in different systems if, if the client has some mental health piece, I get to see that because I mean, if the client has an addiction issue on, I get to see that because I work between the two teams, I can see 'Oh, this client is also being counselled by an addiction counsellor for current disorder.' And yeah, so that's how the client is sort of, the needs, are the unique need of that plan is, is met through the diversity of, of the team and is the skill set that is, is on."*	An experienced workforce plays a crucial role in facilitating integrated care within a system that lacks formal coordination, as their familiarity with services enables them to navigate fragmented structures effectively. Without staff working across both mental health and addiction teams, it is often unclear that a client is receiving support from both, highlighting the reliance on informal knowledge to bridge systemic gaps.	This is dependent on a workforce with experience in a fractured system, leading to their ability to identify and navigate around hurdles caused by siloed working	Understanding that, in the absence of formal integration, care relies heavily on the skills, experience, and informal efforts of providers who bridge system gaps to address clients' complex and overlapping needs.	Critical role of a skilled and adaptable workforce in compensating for structural fragmentation. Integration, when it happens, is often improvised—driven by the provider's initiative and moral commitment—highlighting that without this human element, the system's rigidity would leave many clients without meaningful support.
The participant in this dialogue strongly alluded to how service providers sometimes create unofficial solutions to bypass systemic restrictions and provide necessary care.	*"Yhey do attend our mic drop-in groups that I conduct because it's a drop-in. Anybody can come in. So, all I do is just listen to what they are saying and I'm like, OK, urgent care, go to Saint Paul's hospital, go to find a GP. So that's*	It is plausible to claim that service providers navigate bureaucratic constraints by developing informal solutions to meet client needs, even when these solutions fall outside official protocols.	The inferred phenomenon is dependent upon the presence of professional advocacy and a sense of moral obligation, where providers feel compelled to find alternative solutions when the system fails to meet client needs.	See above.	See above.

	that's, that's how much I can do because there is no way that client can be served in the way where a plan that is eligible for the services we provide will have similar access"				
Participant describes the difficulty that clients face when navigating the care routine/care plan set out by the clinic due to unique individual and institutional barriers that migrants often face (E.g., not speaking English)	*"There are clients who are able to manage this system and get from one point to another, but there are some that just said, I can't do this. I'm new to the city. I'm having really hard time getting to where you're sending me so."*	A singular or uniform approach to care may be insufficient for addressing the diverse and often marginal needs of some clients. In such cases, system navigation and access to reliable information become critical resources. It is plausible that clients have a clear understanding of what they need but lack the knowledge of how to access appropriate services or where to go for specific types of support.	This is dependent on the existence of social norms in care practice, and the subsequent establishment of a mainstream model of care, where needs outside of the model are underserved and/or marginalised due to their divergence from the "norm"	Recognising that mainstream care systems operate within a dominant cultural framework that often fails to accommodate the diverse realities, cultural understandings, and structural barriers faced by migrants and refugees.	A one-size-fits-all model of care, grounded in Western norms, can marginalise clients with different cultural or social contexts. This results in disengagement or inability to access services, highlighting the need for more culturally responsive and flexible approaches to mental health care that address both institutional and individual-level barriers.
**1**The participant in this dialogue strongly suggested that cultural misalignment between Western mental health frameworks and refugee experiences creates resistance to care.	*"I mean in terms of, of that population we have not had a successful integration or we haven't even had that conversation before. Everyone is treated with a broad stroke. It doesn't matter. When you, it's not as you were here, you're referred we treat you the same but I now realise that for some clients, they, they would need a, a different way of identifying what we call mental health issues. A good example is a client who is from a different country, new, perception of*	It is plausible to claim that Western, individualistic approaches to mental health care may not align with the expectations and cultural understandings of refugees, leading to disengagement and mistrust of services.	The inferred phenomenon is dependent upon the influence of cultural norms and differing perceptions of mental health, where discussing mental health openly may be culturally inappropriate or misunderstood, creating a barrier to engagement with care.	See above.	See above.

	mental illness is so really different."				
The participant in this dialogue strongly suggested that disruption is caused when services communicate within their own teams but fail to coordinate across different providers.	*"But Duncan House wouldn't tell us anything about the client and we wouldn't tell anything about the clients. There is no exchange of information to support that client. So, if we send a client to Duncan House, they are sent back to us because they have mental health issues. We send them to them for housing, they send them back to us. So the client is kind of flipping between these two, um services."*	It is plausible to claim that a lack of inter-service communication leads to fragmented, inefficient care, where clients are passed between providers without resolution, creating a confusing and frustrating experience akin to a bureaucratic 'ping-pong' effect.	The inferred phenomenon is dependent upon the influence of a market-driven service model, where providers prioritise internal performance targets over cross-service collaboration, reinforcing a competitive rather than integrated approach to care.	See above.	See above.
The participant in this dialogue strongly suggested the importance of viewing clients beyond their immediate mental health needs, considering housing, employment, and other social determinants of mental health	*"But then further discussion would then kind of open up a whole other needs of that client. The client maybe has undocumented records like there are kind of in the system, but they are in, they don't have any documents. They have, they have housing needs, clothing, food. Some of them do not have GPs. There is mental health, but there is there is the metabolic medical conditions that they have."*	It is plausible to claim that refugee mental health care extends beyond initial psychological concerns, encompassing broader social determinants of health such as legal status, housing, and access to basic services. However, the lack of shared data and documentation across services poses a significant challenge to delivering truly integrated care that addresses these interconnected needs effectively.	The inferred phenomenon is dependent on the presence of empathy and a holistic care approach among providers, enabling them to recognise and address the full spectrum of refugees' needs rather than focusing solely on immediate mental health symptoms.	Recognising that effective mental health care for refugees must adopt a holistic, person-centred approach that addresses both social determinants of health and actively involves clients in shaping their care.	Importance of seeing clients as whole individuals with intersecting needs—beyond just mental health—and involving them meaningfully in decisions about their care. A model that combines empathy, advocacy, and shared decision-making not only improves engagement but also lays the foundation for more sustainable and impactful mental health outcomes.
The participant in this dialogue strongly suggested that involving clients in their care decisions nurtures engagement, motivation, and better mental health outcomes.	*"We will have, at least in my role I normally keep on that motivation. Like, let's do this together, right? Let's do. Let's work together to ensure that these needs are met because they are an essential part of the mental health process in, in providing you with all the*	It is plausible to claim that shared decision-making enhances client engagement by making individuals feel heard and understood, thereby promoting a sense of agency in their healthcare journey. This emphasis on collaboration and working together also supports the delivery of integrated	The inferred phenomenon is dependent upon the existence of empowerment and advocacy for patient voices and choices, which reinforce their role as active participants in care rather than passive recipients.	See above.	See above.

	support we can possibly afford to give you."	mental health care, promoting more cohesive and responsive support systems.			

**Ministry of Health**								
AAB				MH				
Exp	*Quote*	Inf	Dis	Exp	*Quote*	Inf	Dis	**Final**
The participant in this dialogue suggested that increased visibility of mental health and addiction issues has led to greater government funding	*" yeah, kind of we're, we're the hub for everything mental health and substance use and overdose and trying to, you know, go through the uh like ask, ask the central government because that's how it works like meet different ministries, ask central perse, *chuckles* you know big money people in the central government for funding for projects or or specific organisations, and so one of the big things that Ministry is doing is trying to like put as many of the funding requests in relation to mental health and substance use and fund the area as, as well as we can and it kind of works, it's it, it increased dramatically in the last few years."*	It is plausible to claim that increased government funding for mental health care is driven more by shifting societal priorities (visibility) and public pressure rather than proactive government-led initiatives.	The inferred phenomenon is dependent upon the influence of societal shifts in understanding, funding, and prioritising mental health, where public opinion and political agendas push governments to allocate resources in response to changing expectations rather than leading the change themselves.	Participant explains how Indigenous peoples were seen as a priority population and therefore were represented in MH policy guidance for MH treatment	*"Both First Nations and urban Indigenous people were seen as a priority population or vulnerable population for both mental health and substance outcomes. And so like we created the, uh, Pathway to Hope document, kind of in 2018 and were kind of it was our guiding document"*	Prioritisation and/or visibility of a given population's needs may be linked to distributing services based on vulnerability	This happens when social welfare and care is designed to support those in need, thus placing the responsibility of defining need on the organisations and their ability to communicate the potential impact of the service on their client base	*Resource allocation is driven by shifting societal priorities and a given issue/group's visibility in the political arena.*<br><br>**Government funding decisions are driven by fluctuating social consciousness around given issues, largely dependent on self-advocacy and political interests. As a result, well-advocated issues that align with political interests are more likely to receive funding from the government.**
The participant in this dialogue suggested a growing perspective that the medicalisation of mental health has not been entirely beneficial, and the next step	*"It's medical thing. So like it's been medicalised. And since then it was kind of coming hand in hand with mental health,*	It is plausible to claim that the shift towards a biopsychosocial model of care (will) has	The inferred phenomenon is dependent upon increasing awareness of alternative	Participant refers to the way mental health is seen in the past and how moral	*" So like original, original think about substance use that it was like a moral flaw, right? And, and therefore should be, you know, like*	Possibly outdated perceptions of mental health care may lead to inappropriate approaches to	This is dependent on an institutional emphasis on systemisation over personalised care, where medical	*Perceptions of mental health and mental health care are shaped by medical tradition. Subsequent decisions are in tension with contemporary perspectives of mental health*

should involve de-medicalising mental health approaches.	*right? So yes, this is a brain, it's, it's a brain thing, right? And brain things are related to mental health. So I think the next step is kind of separating it out or like de-medicalising it, but that haven't happened yet."*	influenced how mental health is understood at a governmental level, prompting discussions on moving beyond a strictly medical approach.	mental health models that emphasise psychological and social factors alongside biological explanations. This shift challenges the dominant medical perspective, promoting doubt about the sufficiency of a brain-focused approach in fully understanding and addressing mental health issues.	improvements led to medicalisation	**chuckles* changed through corrections or like moral improvements and then kind of it started to become a, a common understanding it is a health problem. It's medical thing."*<br><br>*Could be argued that addiction is medicalized which legitimizes need for resources and support but does not address underlying causal factors?*	unique care situations	tradition and process override the nuances of individual need	*and care, which challenge dominant medical knowledge.*<br><br>**Efforts to promote mental health care that addresses the emerging nuances of mental health needs are in tension with medical tradition. The attachment to growingly outdated practices leads to both ineffective care and mounting frustration from the health workforce.**<br><br>**Yes, SDH complex needs not linked to mental health and substance use**
The participant in this dialogue suggested that the absence of a standardised reporting structure leads to inconsistencies in practice and service delivery across different provinces.	*"No, no. Like the, like federal government cannot give the, uh, provincials directions. So, it's not like the, so say there is BC government. And the BC government is funding and providing directions to the BC health authorities. So that's kind of just this reporting relationship, right? Like our ministry tells the presidents of health authorities, you shall do this and this, and here are money for you to do that."*<br><br>*(note when comes to refugees they are funded federally*	It is plausible to claim that the lack of a structured delivery model means that while services receive funding and general instructions, the absence of centralised oversight leads to decision-making that may not always align with the goals of integrated mental health care.	The inferred phenomenon is dependent upon the presence of confusion and decentralised autonomy, where despite receiving funding and directives, the lack of standardised reporting and accountability allows inconsistencies in decision-making and gaps in integrated mental health care to persist.	Participant describes the funding relationships between different governmental bodies, but the absence of accountability mechanisms between the authorities for mental health and addictions subdivisions	*"The BC government is funding and providing directions to the BC health authorities. So that's kind of just this reporting relationship, right? Like our ministry tells the presidents of health authorities, you shall do this and this, and here are money for you to do that. That, that's kind of it's pretty clear there is no, there is no reporting relationship between Federal Minister of Health and there is no federal minister, mental health, addictions and and provincial minister, right? So they can exchange letters, ask for things and*	Without reporting mechanisms, there is no funder-grantee relationships become simply reporting-oriented	This happens when reporting mechanisms exist to communicate their success in meeting a pre-discussed goal, rather than reporting the impact and nuances of the programme implemented using governmental funding	*Absence of standardised reporting mechanisms that speak across government bodies that go beyond surface level interest in programmatic outcomes may hinder the depth of understanding that gov officials may have over the wider system and its operations*<br><br>**Decentralised autonomy and lack of standardised reporting mechanisms detracts from both a deeper understanding of how disintegrated care impacts the mental health care system, and the possibilities that funded integrated care strategies could have on improving the system.**

	and have received budget cuts)				*inform whichever things, but there is no clear kind of, you know...reporting structure in there"*			
The participant in this dialogue mentioned that the limited availability of trained professionals, particularly those who can provide services in multiple languages, hinders the successful implementation of mental health initiatives.<br><br>Training/knowledge/expertise came up in other interviews	*"There is still a problem of the workforce and there's still a problem of the language, right? So, even if you were spending lots of money on uh, and, and, and especially like I, I think acute in the health, in the health sector, right? So, like you can, you can be willing to spend lot, lots of money on health or people in your province, you can recognise mental health. And, and or substance use as a priority is, and you're willing to pay for it. They're still lack of people who are providing these services. There is, will still be lack of people running services and it will be especially hard providing the services in like in the languages of the refugees. And then the services need to be provided by the certified specialists and we're like getting into this workforce territory and it's really hard, right?".*	It is plausible to claim that language barriers in staff recruitment hinder access or quality of integrated mental health care, as effective communication allows clients to express concerns more openly and with greater ease.	The inferred phenomenon is dependent upon the role of connection and trust in healthcare provision, where speaking in a foreign language may lead to confusion, mistrust, or alienation, ultimately reducing engagement and limiting opportunities for shared decision-making.	When funding is provided, nothing happens because there is not enough personnel to run the programme	*"So, like you can, you can be willing to spend lot, lots of money on health or people in your province, you can recognise mental health. And, and or substance use as a priority is, and you're willing to pay for it. They're still lack of people who are providing these services."*	Funding becomes irrelevant when there is no one available to run the programme	This is dependent on the systems need for specialised support, coupled with the limited number of trained experts available per province, leading to a shortage of human resources in each provincial system	*Forced migrants have unique needs that not all care providers can meet, leaving a gap in care even if funding is provided.*<br><br>**The centrality of shared language and culture between providers and clients shapes the high demand for specialised linguistic services, yet there is a low supply of that service, leading to a persisting gap in care.**
The participant in this dialogue highlighted that investing in early intervention is seen as a long-term strategy to reduce the prevalence of mental health and substance use issues.<br><br>Implementation of early intervention strategies can decrease governmental spending long term	*"Because like the idea is that when you start early then it prevents, you know, development of mental health or, or like mental health related conditions or substance use conditions, right? And so ultimately you don't run into more problem down the road."* MT, Line 281-284<br><br>Resonates with earlier data where refugees are seen in crisis; blamed as individual level response delayed	It is plausible to claim that the emphasis on long-term prevention strategies reflects a shift away from traditional, medical-paternalistic? models of care toward a proactive, prevention-focused approach that prioritises early	The inferred phenomenon is dependent upon growing awareness of the complex and fluctuating nature of mental health trajectories, particularly among refugees. This recognition supports the transition from reactive (crisis)to proactive service	Participant defines the importance of prevention, and the subsequent interest in serving children's mental health	*"Prevention is a reason, reason for why children and youth are kind of chosen as a population, right? Because like the idea is that when you start early then it prevents, you know, development of mental health or, or like mental health related conditions or substance use conditions, right?"*	Population prioritisation is driven by risk of disease development and a life course rationale, leading to specific populations such as children, being emphasised in prioritisation	This is dependent on normative definitions of vulnerability, and the capacity for organisations and individuals to be deemed vulnerable based on specific criteria	*Prevention is a priority strategy in the healthcare workforce, with the goal of reducing system demand and long-term illness.*<br><br>**Strategic emphasis on prevention leads to interest in early treatment, lending to possible investment in early intervention and indicates funder support for proactive programming**

	access to care may be systemic problem	intervention over crisis management.	provision, aiming to minimise the long-term impact(outcome) of poor mental health.					
The participant in this dialogue suggested that the involvement of multiple government ministries, health authorities, and community organisations creates a fragmented landscape that requires collaboration to navigate effectively.	*"It kind of even with the provincial government or province kind of taking responsibility for all things health like it's, it's still this like days the government, multiple ministries and government both Ministry of Health and National Health, additional health for regional health authorities, FNHA, a community organisations so it's a complex landscape and definitely like it's, it's, it's about everybody working together and figuring things out."*	It is plausible to claim that greater collaboration and integration between different agencies (ministries) could improve refugee mental health care delivery (collaboration) by reducing fragmentation and creating a more cohesive system rather than multiple disconnected structures.	The inferred phenomenon is dependent upon the presence of shared responsibility and a collective willingness to prioritise collaboration over political and organisational agendas. Effective service integration requires a commitment to working as a unified system rather than operating in silos.	Participant describes the importance of cross-agency collaboration and communication as the key to integrated teams. He emphasises the importance of information, including data and case work as particularly important units of comms to promote unified care for a single patient	*"So kind of the I think for the integrated teams, it needs to be. There is a need for information sharing between different agencies and, and different types of specialists within the team. There is a need for kind of team integration so there is a psychiatrist and a nurse and a social worker that are kind of physically collocated. And then can work with clients as a team. Both sharing data among themselves and then coordinating their efforts and being able to come together, talk to client as a team and then take cases back to their kind of respective agencies"*	Case management across organisations is enabled by transparent and consistent communication between agencies	This is dependent on the providers willingness to take on the extra labour associated with overcoming the existing communication barriers, and willingly adding extra work to their already strained schedule	*Shared interest in overcoming organisational siloes and promoting transparency, communication and collaboration between agencies is present. However, the commitment required to orchestrate this collaboration is limited by the multiple and fragmented governmental influences.*<br><br>**Although there is great interest in integration, government regulation is managed in a fractured way. This fragmentation translates into the "chain of command", where the ability of agencies to commit to integration activities is hindered by the (dis)organisation of higher institutional operations.**

**Specialized Refugee Primary Care Clinic**								
AAB				MH				
Exp	*Quote*	Inf	Dis	Exp	*Quote*	Inf	Dis	**Final**
Participants expressed how pre-existing legal classifications, and bureaucratic requirements determine whether refugees can access	*"So that the challenge is if you were a different if you're not a GAR or a privately sponsored refugee you don't necessarily have access to the same*	It is plausible to claim that the bureaucratic process is likely to create restriction in the way integrated mental health care is	The inferred phenomenon is dependent upon the existence of institutional tendency to enforce rigid professional boundaries, which	GARs and PSRs receive different resources, leading to very different treatment outcomes. With GARs subsiding, participants suggest that individuals that	*Now that we're seeing less GARs this settlement. I met a lovely patient yesterday and speaks English. She was from Afghanistan, a government assisted refugee, and she just, she said, oh, she's found her voice and she's she can*	Refugees' sponsor may play a central role in defining the level of support one receives, suggesting that settlement outcomes may differ massively if you are not a GAR or PSR	This is dependent on the legal preface which outlines refugee care that providers must follow, where providers are unable to allocate resources for refugees that fall outside the remit of either the refugees' resources and/or	*Pre-existing institutional processes and stipulations restricts the possibilities of integrated care delivery by limiting access to resources based on a labelling system.*

care, often creating arbitrary barriers.	*settlement resources, so that same kind of orientation classes or newcomer centre, family centre if you're a sponsored mom, a sponsored woman who comes here and she's pregnant under the age of 25, you can't access the family centre because you're technically a sponsored immigrant versus a refugee”*	delivered and accessed	can cause fear of consequences.	fall outside the range of GAR and PSR will not have the same level of support	*speak up for herself. And I said how, what helped you to find that confidence? She said, well my settlement worker sent me to a leadership workshop, and I learned that in Canada I can speak my mind and whatnot. So that the challenge is if you were a different if you're not a GAR or a privately sponsored refugee you don't necessarily have access to the same settlement.*		the pre-determined treatment path	**Government labelling and sponsorship sources leads to variations in available resources based on the label/sponsor, ultimately determining the treatment and level of support possibilities despite care needs.**
It is plausible to claim that the label assigned to refugees upon entry into Canada acts as a facilitator or barrier to accessing services, resulting in inequitable access to care based on sponsorship type rather than individual need.	*“And they're kind of just linked into the settlement system a little bit more effectively and link classes, English classes more effectively than the PSRs. Their sponsors don't necessarily help them to get into English classes. They really want them to start working. And so that's kind of problematic.”*	The inferred phenomenon (integrated care) is dependent upon a bureaucratic system that imposes Labels which categorize who has access to care creating barriers for healthcare providers, restricting their ability to make care decisions based on client needs rather than institutional policies.	The shrinking service system is leading to increased patient load at [clinic], and now they're seeing clients they would not have otherwise had (privately sponsored vs GARs) Postulating theory: services become less integrated when mandates/policies are guided by how people are categorized PSR vs GARs	*We used to have more government assisted refugees as a partner clinic but with the dissolution of bridge clinic that that work being privatized as well as the federal government now increasing privately sponsored numbers versus government assisted hence kind of offloading some of the responsibility to community. We're seeing more privately sponsored and I think that does affect how people settle because they simply don't have the same level of support that government assisted refugees have, right? NCC 143-148*	Privately sponsored refugees face different challenges in the system, . This suggests that the services available may not be fully utilised because PSRs are unable to access them, placing greater responsibility on individuals and not the health care system (neoliberal/fiscal restraint lean funding) overworking a specific arm of the settlement system	This happens when the resources allocated by the government are influenced by political and public discourses on who has access to resources e.g. anti-immigration politics placing greater responsibility on the public sector to support privately sponsored refugees Changing the dynamics of the kind of support offered	Participants highlighted the differences between government-assisted and privately sponsored refugees, including issues of dependence, , and unequal access to services.	
Participants shared the impact of high referral rates, limited resources, and overwhelmed	*“Yeah, so after they get discharged. Yeah, unfortunately, some of them end*	It is plausible to claim that the existing pressures within healthcare services and lack	The inferred phenomenon is dependent upon the presence of a resource-strained	Counsellors attempting to reach patients on a waitlist may not be able to reach them because	*And often like a lot of the counsellors like they may, once they triage the referral after they've been on a wait list for 6-8*	Counsellors managing high volumes of cases may be less inclined to persist through gaps in communication between	This is dependent on the extent to which the counsellor is overworked, where their limited capacity leads them to	*High demand for care coupled with cultural differences in how health care is delivered can lead to inefficient*

clinics on the availability and timeliness of care.<br><br>Connects with previous themes of waiting for crisis	*up just using walking clinics or urgent care. Some find GPs and to maybe a handful we try to assist them in finding a GP, but we just need to do that because we need ongoing capacity basically because we get probably 20 referrals a week on average."*	of funding support for increase staff increase the likelihood of burnout and disengagement as capacity and resource are mismatched, ultimately leading to negative outcomes for both providers and service users.	and high-stress healthcare environment, where chronic understaffing and systemic instability generate frustration, , and staff burnout .	patient/refugees are busy working or in school; there are cultural differences in how health and social care is accessed from country of origin, e.g. drop in<br>Not being able to reach patients causes lack of follow up support	*months. If they give them a call once and they cannot reach them they close their referral, right? So a lot of chasing with that.*	agencies that exist due to limited human resources, leading to pre-emptive closing of a given case	prioritise other professional duties over persisting one specific case<br>Postulating theory:<br>There is a cultural mismatch between how refugees access services in country of origin vs Canadian context where you can be reached by phone to make an appointment. Cultural practice in Canada is dependent upon you being available and making an appointment for counselling.	*care.an eager workforce and a poorly resourced system leads to workforce burnout.*<br><br>**Systemic instability and chronic resource limitations lead to overwhelm and burnout amongst a deeply committed workforce and subsequently cut corners and or limited engagement in all parts of the treatment cycle. This can lead to clients not receiving care.**
Participants identified the consequences of disjointed healthcare services, lack of communication, and poor information-sharing on care coordination.	*"We send a referral. They call them three times and they send us notes saying we weren't able to get a hold of them. The file is closed and I've literally called that same case manager up and said he got your call. He tried to call you back, you didn't answer. And so it's the circular kind of way of trying to get into mental health, which is not, which is not perfect, and then wait times. So an urgent referral that was sent two months ago, patient is being seen by psychiatry in August for a chronically suicidal gentleman. The wait times and and the route to get in is not great"*	It is plausible to claim that frontline service providers bear the brunt of systemic failures, as they attempt to navigate and work within restrictive and fragmented healthcare structures, often unable to provide timely and effective care due to bureaucratic inefficiencies.	The inferred phenomenon is dependent upon the presence of widespread frustration toward a system that lacks integration, where delays in information-sharing and poor coordination impede staff from doing their work efficiently—directly affecting service users who experience the consequences of these systemic barriers.	Participant describes the labour involved in conducting referrals across different types of organisations and the processes they encounter when attempting to refer	*I had to call myself RCH, when I refer to one place, there are no, no, no, we're not reproductive. Call this place. I had to call that place. They had to tell me. Give me. Send me the referral forms like fill it out, fax it just to find that out*	When working with organisations with different care mandates, providers may need to navigate through several steps to appropriately refer their patients	This is dependent on resources and direct pathways to care and specialized services the persistence and knowledge of the provider, who would need to be motivated to take on the labour and skilfully adhere to multiple bureaucratic processes of different organisations	*A fragmented system burdens itself on healthcare workers by requiring multiple and even contradictory bureaucratic hurdles. Providers are then forced to navigate and exercise ad hoc remedies to the fractured system with limited support.*<br><br>**A chronically fractured care ecosystem forces care workers to navigate multiple and separate organisational processes and disjointed information sharing. This can leads to frustration and fatigue, but ultimately requires all staff involved in the system to be capable of navigating this complex ecosystem with a certain willingness to overcome fatigue.**

**1.5.** Participants highlighted how refugees' cultural backgrounds shape their perceptions of mental health, often leading to scepticism or reluctance to engage with Western-style services.	*"And it is like for some of them, yeah, they they're not very open to it. They're like, no, I don't want it. Some of them like, Ohh, they use an interrupter right? Like yeah, they use a for interpreter you can not disclose your name or anything. There is a stigma attached to it and some people not open to the idea of counselling."*	1.5 It is plausible to claim that Western, individualistic approaches to mental health care may not align with the expectations and cultural understandings of refugees, leading to disengagement and mistrust of services.	1.5 The inferred phenomenon is dependent upon the existence of influence of cultural norms and differing perceptions of mental health, where discussing mental health openly may be culturally inappropriate or misunderstood, creating a barrier to engagement with care.	Stigma around counselling and the use of an interpreter might lead to hesitance to engage with counselling	*Cultural, yeah. And it is like for some of them, yeah, they they're not very open to it. They're like, no, I don't want it. Some of them like, Ohh, they use an interrupter right? Like yeah, they use a for interpreter you can not disclose your name or anything. There is a stigma attached to it and some people not open to the idea of counselling.*	Stigma plays a role in a patient's willingness to engage with the care system, which can be further disincentivised when a patient perceives high risk for exposure	This happens when clients feel pressure from their communities/stigma around mental health, but are in enough need of support to seek care	*Different perceptions and expectations of mental health care may cause refugees to hesitate before approaching the care system.*<br><br>**Stigma and social norms around mental health and mental health care may lead to mistrust and/or hesitance in refugee communities around engaging the care system. Without sensitive and humble care, those who need it most from these communities may not feel safe engaging the system.**
Participants observed the impact of digital barriers, privacy concerns, and lack of infrastructure on refugees' ability to access online care.	*"Because a lot of patients can access those on an app, on their phone, be a part of group, right? The English speaking. And I know they exist. But how appropriate is it for patients that are refugees? They may not be able to navigate systems. You know, technology that way or they may not even I've had patients that they found the group counselling online, but they said I have no privacy at home to do it. How can I find a space to zoom in and tune into this CBT they're offering? For example, right?"*	It is plausible to claim that inequitable access to digital technology, coupled with a lack of private and safe spaces for online engagement, creates barriers that make it more difficult for refugees to access digital mental health services.	The inferred phenomenon is dependent upon pre-existing disparities in refugee circumstances, where differences in digital literacy, access to reliable internet, and safe spaces for engagement directly influence their ability to participate in online care.	Although app-based access points is known to help promote healthcare access, refugees may not have the same experience due to differences in language, access to mobile phones and familiarity with the system	*Because a lot of patients can access those on an app, on their phone, be a part of group, right? The English speaking. And I know they exist. But how appropriate is it for patients that are refugees?*	Existing methods that help clients manage the complex care system may not be as accessible to refugees, making care inaccessible	This happens when clients do not have electronic devices and/or cannot navigate apps due to language barriers and/or literacy of the new system in the host country	*Differences in access to digital health services and/or linguistic differences makes it harder for refugees to access care in ways that generally aid other local clients.*<br><br>**Access to digital technologies in Canada vary for refugees, where content and/or services are mainly provided in English. This limits access to alternative mental health care outlets for refugees.**

Participants expressed wishful thinking about having a system that has good communication, integrated systems, and accountability.	*"I wish we had a closer like a bat phone to them or a different route of referral process. Like if we had a complicated person with psychosis not yet defined or whatnot that we could. That we just had a closer relationship. With the New West and I feel like New West Health works better. I don't know why."*	It is plausible to claim that service providers are seeking more integrated systems and closer relationships with other providers to facilitate seamless care and better service coordination.	The inferred phenomenon is dependent upon the presence of advocacy and a well-intentioned vision for improvement, where despite existing challenges, service providers hold onto the hope that stronger communication and integration could create a more effective and responsive system.	Participant describes the impact that a more complex disorder has on their decision-making, namely their need for external input from other experts, leading them to seek out information from other members of the service system	*Then our doctor, you know, that was seeing him like, OK, I called RACC line, and they make some vague recommendation. But really, we wanted them to see the patient cause their presentation was like OK bit of paranoia, schizophrenia. But this mood disorder now that you're mixing in, right? We may not feel comfortable.*	Providers may be more inclined to seek support from other organisations when they believe their expertise is insufficient to address a complex case	This is dependent on providers trusting and relying on external support to effectively address complex needs beyond their knowledge	*Care needs are not straight forward, and service providers may require aid from other corners of the care ecosystem.*<br><br>**Service providers must lean on one another to deliver holistic and effective care when attempting to meet the complex care needs of refugee clients.** .
			Participant expresses uncertainty around the availability of external specialised services that are important for their patients, yet inconsistently available.	*I don't always have my finger on the pulse of what's happening there. Like has their funding shifted? Are they capped out in terms of referrals? Referrals goes out online and nothing comes back. And so we really don't know. So it's such an important resource for us and yet I never know what their capacity is and it's easy to be hit and miss whether people are seen.*	Providers may be keen to establish connections with other organisations, however other organisations may not have the capacity to connect nor the ability to communicate their capacity	This is dependent on providers faith in other organisations' support, and the capacity of the other organisation to promote integration	Participant expresses uncertainty around the availability of external specialised services that are important for their patients, yet inconsistently available.	

Survivor Advocacy Group					
**Experiential**	**Quote**	**Inferential**	**Dispositional**	**Consensus**	**Final**
Participant who is a provider with lived experience explains that [organization] workshops and trainings were very empowering compared to	*"I found I participated in some of the trainings and workshops that [THE ORGANIZATION] offered. I found it super empowering*	Holistic attention to the social determinants of health enables programmes to empower clients, which	This is dependent on the complex needs of refugee clients, and the impact that mental health has on the settlement process which	Recognising that holistic, multi-dimensional support—encompassing mental health, advocacy, and social needs—promotes	When services move beyond narrowly defined mental health support to include social, legal, and

other agencies because they provide a holistic set of services such as mental health and advocacy.	*comparing to others to other agencies, both in British Columbia and the Wisconsin State, maybe I think that's the and it's also they look at the mental health from a holistic perspective, not just specifically and narrowly focusing on mental health that ohh, OK, you know, we provide you these services and that's all we will do. They do lots of advocacy for immigrants and refugees. They do advocacy in provincial and federal level. They empower their clients and also the the services that the clients and beneficiaries of [the organization] receives empower the client directly but also helps the rest of the family or the loved ones of the clients to feel empowered." L77-85*	leads to more effective mental health care	impacts every corner of a refugee's life<br>Thinking beyond biomedical frameworks can help address social determinants of mental health for refugee clients; this includes holistic view of client and their families.	deeper engagement, empowerment, and trust among refugee clients.	familial considerations, they are better equipped to meet the complex realities of refugee life. This approach not only empowers individuals but strengthens community wellbeing, making care more effective and sustainable.
Participants described when services consider social, legal, and advocacy needs alongside mental health care, it enhances engagement and trust.	*"Their holistic approach of going beyond, you know, they're brave enough to go beyond mental health needs of refugees and immigrants. They do advocacy. They help them with sometimes with settlements. They provide services like, for example, because the services was useful for me I encouraged my wife to come and get services from that. Now she's getting two different types of services from [the organization]. One is mental health, the other is social kind of social support and also some some sort of*	It is plausible to claim that refugee mental health care extends beyond initial psychological concerns, encompassing broader social determinants of health such as legal status, housing, and access to basic services when SDH are addressed it fosters trust.	The inferred phenomenon is dependent on the presence of a holistic care approach which acknowledges not only the individual but family which enables providers to provide integrated care whilst recognising and addressing the full spectrum of refugees' needs rather than focusing solely on immediate mental health symptoms.	See above.	See above.

	vocational support is offered too."				
Participant discusses the realities of mental health seeking behaviour, highlighting the importance of being present when clients are ready to approach services because clients may not feel the need for services right away	*"I also work with newcomers and at the beginning when you mentioned the resources like they don't say yes to the resources, but sometimes later on they come to they're like, oh, yeah, I think I could use that resource. Like, can you refer me or can you help me with this? So I can just mentioning what is there and also I think there are some things ensuring that there's going to be a safe space"*	It is suggested that providers must meet clients where they are to ensure that clients feel safe and ready to receive care despite their need for it	This is dependent on the complex nature of trauma and mental health care seeking behaviour that might contradict perceived urgency for treatment	Timing and readiness are crucial in refugee mental health care, as clients may not engage with services immediately but respond better when approached at a pace aligned with their settlement journey and emotional capacity.	Effective care requires providers to be patient, flexible, and attuned to when clients are ready to receive support. Trauma, resettlement stress, and personal readiness mean that service uptake often occurs gradually—care that respects this timing promotes trust, safety, and sustained engagement.
Participants in this study described the importance of providing support at the right time until refugees are settled to avoid overwhelming them	*"Yes, it's more of a yeah training program I would say. It's also like for people who are a little bit have been here for a longer time for like a bit like settled with the refugee journey. You know, if because that period can be a stressful time and then it's offered for them."*	It is plausible to claim that the timing of support is crucial, as providing care when refugees are ready increases the likelihood of engagement and meaningful participation in services.	The inferred phenomenon is dependent upon the presence of cognitive overload during the early stages of resettlement, where refugees must navigate multiple stressors. If support is introduced too soon or at the wrong time, it risks overwhelming them and reducing engagement.		See above.
Participant explains that for providers who share community with their counselling, it is especially important to explicitly define confidentiality so that the client is not concerned that the provider will discuss their case with other members of the community.	*"Although they are from could be people could be from your community but they still they have to abide by the confidentiality rule like they cannot go to talk around the community like it's going to still be like confidential. I think things like that would be, at least for myself, like as soon as someone says, like can say everything with me and it's going to be within this space. And I'm not gonna go around. I think that's was a*	The promise of client confidentiality may be broken when provider and client are from same background which may threaten the patient/provider relationship.	This is dependent on a client's perceived risk, trust in the system and fear of stigmatisation associated with mental health care and/or ill mental health	Recognising that in communities where stigma around mental health is high, especially when clients and providers share cultural or community ties, explicitly assuring confidentiality is essential to promoting trust and openness.	Confidentiality is not just a procedural requirement but a culturally sensitive practice that can make or break engagement in mental health care. When clients feel safe that their disclosures won't circulate within their community, they are more likely to share openly and begin meaningful therapeutic work.

	safe. Like it's gonna, like, allow me to speak to my experience and not be worried about how to filter it or what to say what not to say"				
Participants expressed how assurances of confidentiality encourage refugees from stigmatised backgrounds to disclose their struggles without fear.	*"OK, they're going to know us. We are from Afghanistan, you know, like this story, this it's going to be like. Kind of saying that like although they are from could be people could be from your community but they still they have to abide by the confidentiality rule like they cannot go to talk around the community like it's going to still be like confidential. I think things like that would be, at least for myself, like as soon as someone says, like can say everything with me and it's going to be within this space. And I'm not gonna go around. I think that's was a safe. Like it's gonna, like, allow me to speak to my experience and not be worried about how to filter it or what to say what not to say"*	It is plausible to claim that in some refugee communities where mental health is stigmatised confidentiality must be upheld to promote cultural safety	The inferred phenomenon is dependent upon the presence of fear and perceived repercussions associated with disclosing mental health struggles in Afghan communities, where stigma can discourage individuals from seeking support unless confidentiality is assured.		See above.
Participant explains that counsellors with shared language can help improve patient comfort because there may be more pathways for patient/provider connection and/or the room is not full of other interpreters	*"I think ideally if the counsellor speaks your language, I think that's much easier because then you don't have to worry about the second, third person like in the room or also maybe you have a psychological connection or something. "*	Linguistically appropriate care may help improve effective care because it can accentuate the personal connection established by patients and providers during treatment	This is dependent on the provider and patient's inability to communicate with one another, which not only limits care quality but also diminishes the ability of each party to convey complexity and establish shared understanding	Shared language between provider and client not only reduces practical communication barriers but also promotes emotional safety, connection, and trust—key components in effective mental health care.	Linguistically appropriate care enhances client comfort by removing the need for interpreters and supporting a more personal, direct therapeutic relationship. In refugee mental health contexts, this shared language becomes a gateway to deeper engagement, reducing

					barriers to disclosure and promoting a stronger sense of belonging and trust in services.
Participants shared the role of shared language in nurturing belonging and trust, while the lack of it hinders access and engagement with services.	*"And I think ideally if the counsellor speaks your language, I think that's much more easier because then you don't have to worry about the second, third person like in the room or also maybe you have a psychological connection or something. Because I'm saying that I had my mom for that for like I referred her to another organization in Victoria. And then and someone suggested that, but there's a specialist that it's sometimes it's easier for people to connect with the people who speak the language for this kind of services. But of course, that's not always the situation if that's going to be considered, that's I think it's going to be helpful."*	It is plausible to claim that language serves as a crucial facilitator in integrated mental health care, as it provides a sense of safety, reduces reliance on third-party interpreters, and promotes trust between service users and providers.	The inferred phenomenon is dependent upon the fundamental human need for safety and connection, where a shared language acts as an initial trigger for refugees to feel comfortable enough to disclose concerns and engage with services.	See above	See above
Participant describes the role of the cultural broker, and the importance that they have in helping clients navigate multiple resources to prevent clients from feeling overwhelmed or lost	*"So, I think for cultural broker, it's more, it's a more direct support for the client. If there's a gap in between that client cannot navigate it by themselves, the cultural broker would be a resource to help them to navigate that"*	Cultural brokers play a special role in fostering patients' perceived support when navigating a multi-faceted care system	Collapse with 5.16	Cultural brokers serve as essential mediators who bridge the gap between complex service systems and the cultural differences of refugee clients and western health care system, helping to reduce and promote access to care and services, i.e., mitigate system barriers.	Cultural brokers play a vital role in promoting trust, navigating systemic barriers, and ensuring care feels relevant and respectful to clients' cultural contexts. By guiding clients through services and advocating for culturally sensitive approaches, cultural brokers can enhance both access and engagement in mental health support.

Participants described the importance of the culture broker and designing services that align with refugees' cultural perspectives to avoid alienation, medicalisation, or distrust.	*"What I have known from my perspective for the culture broker, that's a shared program that is funded by the Government of BC and we work with collaboration with MOSAIC and ISS of BC at the beginning that [THE ORGANIZATION] was creating that program was we had three collaboration meetings with Afghans because that's specifically for Afghan participants. We we have talked to different groups of people about, like, what kind of topics that we need to include the program, what we need to be careful and how to like create that program. This is one thing that we have in the culture broker for the mental health part in the intake things."*	It is plausible to claim that a culture broker serves as a mediator, navigating the complexities of cultural differences to facilitate culturally sensitive care. When people with lived experience are included in direct service provision/policy health care services can be better informed about community needs	The inferred phenomenon is dependent upon the presence of deep-rooted cultural norms and beliefs that exist independently of service provision. To promote engagement, services must acknowledge and address these norms rather than operate as if they do not exist. When consultation is sought from refugee communities, it not only enhances the relevance of programs but also supports the inclusion of a cross-cultural broker role. This role is strengthened through meaningful community consultation, which in turn promotes the empowerment of community groups.	See above	See above
Participant expresses concern over the absence of culturally informed care, and the impact that this has on care quality. Specifically, the disconnect experienced in the clinic, and how this leads to poor quality care and even frustrating care.	*"Sometimes even the counselling sessions are not trauma and not culturally informed. And I have this experience. I receive services from other organizations which the counsellor the techniques and the way have they communicate or the techniques that they are using is, is not something that you can connect with, and I've heard that a lot from the clients as well, and sometimes even they get angry"*	In the absence of culturally responsive care, providers may be unable to foster a sense of connection with their clients, leading to less effective care	This is dependent on whether there is perceived distance between the provider and patient that interferes with the patient's ability to trust and therefore confide in a provider, and whether or not a patient feels overwhelmed by the complexity of the system	Lack of culturally informed techniques/interventions rooted in western frameworks care creates disconnects between clients and providers, reducing the effectiveness of therapeutic techniques and potentially causing frustration or disengagement.	When mental health care does not reflect or respect a client's cultural background, it risks being perceived as irrelevant or even alienating. Culturally unresponsive care undermines trust and connection—essential components for effective therapy—reinforcing the need for services to integrate culturally grounded approaches that resonate with refugee clients' lived experiences.
Participants highlighted how the misalignment between	*"Like and you might have techniques to, that might be*	It is plausible to claim that cross-cultural brokers can be	The inferred phenomenon is dependent upon the existence	See above	See above

Western individualistic mental health approaches and community-driven refugee perspectives, leading to disengagement.	*very helpful to clients, and you know that, but that's important that your client feel connected to that. If they're not feeling connected so that doesn't work and trust is important. Like you have to know them, you have to know their culture. You have to respect and so then that they can trust first. And later you can provide that techniques and I think in Canada it happens a lot that the, the counseling is not culturally informed. And even it happens to me that I'm somehow I'm trying to mention that that this is doesn't work. But here I feel sometime people who study here, they thinks that those who are coming from other countries, they might not know um like us. I'm think, what I've learned is different and more accurate. Yeah, and I'm happy that we have the control broker. Yeah, we have lots of things that we need to work on and we improve that, but I hope we can have these things in the, the counselling things as well."*	instrumental in helping refugee clients connect to health services, while also providing guidance and consultation to Western-trained counsellors. This is particularly important given that individualistic approaches to mental health care may not align with the expectations and cultural understandings of refugees, often resulting in disengagement and mistrust of services.	of influence of cultural norms and differing perceptions of mental health, where discussing mental health openly may be culturally inappropriate or misunderstood, creating a barrier to engagement with care.		
Participant is frustrated with the homogenisation that migrants face in Canada, and how the assumptions born from perceived homogeneity leads to care that is not necessarily responsive to the individual, rather it is responsive to an assumption	*"Although we are coming from the same country, but like every ethnic group or every person within that country could have a very different experience, because the way the system was there. So I think that's very important to recognize*	A provider who is unfamiliar with the sociopolitical push factors that different refugee groups face may overlook the complexities of their experiences by treating patients through a preconceived lens,	This happens when refugees are perceived in a specific way and when providers are unaware of the nuances and complexities of the political backdrop that created refugees' migratory situations	Assumptions of homogeneity among refugees can lead to reductive, standardised care that overlooks the diverse and complex socio-political realities shaping individual experiences.	Treating refugees homogenously undermines equitable person-centred care. Effective mental health support must move beyond surface-level identifiers like nationality and instead consider the unique intersections of

	that instead of like trying to have a like generalization of experience within the country. Of course we know that this, that that like there is a war, it impacts the whole population. But there are also some like different programs that impact certain communities that others I think recognizing that it's very important "	ultimately leading to lower quality care. To mitigate this, it is important to acknowledge the differences that exist within refugee cultural groups and adopt an intersectional approach that addresses the diverse and nuanced inequities they experience—for example, recognising that not all refugee groups have trauma histories.			ethnicity, history, and individual trauma. Without this, services risk perpetuating invisibility and offering care that feels irrelevant or even dismissive.
Participants highlighted the risk of assuming homogeneity among refugee groups, leading to one-size-fits-all services that fail to meet diverse needs.	*"I think treating people like with their own experience, you know, recognizing their experience because sometimes, let's say, if you go somewhere and they say like, oh, you're from Afghanistan, like or else have a friend from Afghanistan like, although we are coming from the same country, but like every ethnic group or every person within that country could have a very different experience, because the way the system was there. So I think that's very important to recognize that instead of like trying to have a like generalization of experience within the country."*	It is plausible to claim that standardised, one-size-fits-all mental health services fail to align with the complex and varied experiences of refugees, as they assume shared backgrounds and needs, despite the diverse realities of migration, trauma, and identity.	The inferred phenomenon is dependent upon the presence of stereotypical views and cultural bias, where service providers may attempt to generalise refugee experiences rather than adopting a person-centred approach, despite individuals sharing similar national or ethnic identities.	See above	See above
Participant explains that the lack of collaboration hinders the efficiency of the system, not the lack of staff. Instead, the issue is asynchronized work pacing that leads to asynchronized waitlists, stalling the process at certain agencies	*"and I don't think I now work in the mental health organization and I don't think it's always the lack of like the staff that who provide mental health services, the counsellor. It's lots of time it's the lack of a good collaborating work*	Uncoordinated processes across organisations further accentuate the siloes between services, resulting in prolonged and inconsistent treatment pathways. In previous interviews with care providers, a key theme	This variability is largely driven by organisations operating under their own mandates, goals, and internal processes, which results in differences in efficiency and capacity. Questions arise around whether increasing staffing and resources could	Fragmented and poorly coordinated workflows between organisations—not merely staff shortages—are key drivers of long wait times and system inefficiencies in mental health care.	Structural issue of disjointed collaboration across services, where uncoordinated processes and siloed operations prolong access to care and undermine system trust. Addressing inefficiency requires not just more

	together and here I don't know Sometime I feel like people are not working enough and Like it's not acceptable that we like, for example, we bring excuses that We have, uh, a waiting list of three months, but the other organization have one year. That's not a good excuse"	emerged: refugees are often not seen until they are in crisis. While individual barriers contribute to delays in help-seeking, structural conditions—such as long waitlists and delayed access to mental health support—also play a significant role.	reduce long wait times and enable faster access to care. Ultimately, these issues reflect not only systemic constraints but also the limitations of individual capacity and operational efficiency within services.		resources, but intentional, coordinated collaboration between agencies to ensure smoother, more consistent pathways for both clients and providers.
Participants expressed how long waiting times, lack of integration, and stretched resources frustrate both providers and clients, reducing trust in the system.	*"Overall, for health is the long waiting list for the, the mental health services. This, for example, it happens that I I know about other organization as well. There is a waiting list of more than a year. I myself requested to for two other organization that they provide mental health services. And one of them even did the intake with me, but it's more than one year that there is no response from them and I don't think I now work in the mental health organization and I don't think it's always the lack of like the staff that who provide mental health services, the counselor. It's lots of time it's the lack of a good collaborating work together"*	It is plausible to claim that the existing pressures within healthcare services increase the likelihood of long waiting times and disengagement, ultimately leading to negative outcomes for both providers and service users.	The inferred phenomenon is dependent upon the presence of a resource-strained and high-stress healthcare environment, where chronic understaffing and poor communication generate frustration, helplessness, and disengagement among all stakeholders.	See above	See above
The realities of treatment needs contradict the way treatment resources are allocated to clients. The extent to which you have access to appropriate care that is aligned with your needs is shaped by the origins of your funding and your status	*". And I think another issue is with the funding with the newcomers especially they have an it's called IFH that covers only for the first year. But we know that sometimes for people it takes many, many years to go to that services, so like you are*	The realities of patient needs are in tension with the way the system is structured. This leads to a misalignment in resource allocation: when patients do not need the resource, the resource is available, when they need it, it is no longer available	These challenges are rooted in the rigidity of existing systems, which often struggle to accommodate the nuanced and evolving nature of mental health care. Compounding this is the reliance on short-term funding for mental health programs, driven by	Acknowledging that systemic rigidity and funding structures often fail to align with the long-term and evolving needs of refugees, creating gaps in care precisely when support is most needed.	Troubling misalignment between administrative frameworks and the lived realities of refugee clients, where access to care is shaped more by funding timelines and eligibility criteria than by actual need. This results in care

	providing that support, that insurance that you can use it for a year, but what happens after a year like are you assuming that everything will be fixed put together in one year or is it one year journey? And then you're all settled in Canada, kind of thing"		fiscal restraint, despite the reality that mental health issues—particularly trauma—often emerge over time and are intensified by the ongoing stresses of resettlement.		that is time-limited, conditional, and ultimately ill-suited to the non-linear nature of mental health recovery
Participants described how predefined refugee classifications influence access to care, creating restrictions based on administrative criteria rather than need.	*"Um yeah so and then people, it's mostly internal referral. Yeah so like they fill out, there's a criteria. There's an application they say like, OK, if you have connection with [THE ORGANIZATION], if you have gone through the refugee claiming process, you are protected person. And then you have like the English requirement because it's going to be offered in English. You have connection to Internet and things like that"*	It is plausible to claim that system-imposed restrictions contribute to delays, frustration, and a bureaucratic 'tick-box' approach.<br><br>This theme relates to previous themes about labelling and legal process for refugees who is intitled to what based on how refugees are categorized (sponsored vs government assisted , asylum claim etc)	The inferred phenomenon is dependent upon the existence of a bureaucratic system that imposes rigid administrative boundaries on healthcare providers, restricting their ability to make care decisions based on client needs rather than institutional policies.	See above	See above
Participant highlights the importance of connecting with the client over implementing a counselling strategy, underscoring the importance of trust in effective treatment and P/P relationships	*"sometimes it's also a good idea to like you offer program with intention of like mental health support, but they should not be like highlighted, you know like find a way that you can still offer the support. It could be like maybe getting together at a park, an example would be like that would still bring the communities together, they could speak their language, things like that. Because sometimes, like just naming it mental health*	It is suggested that the connection between patient and provider is more important than strict adherence to traditional models of care, and this connection can be promoted in diverse ways. This aligns with evidence highlighting social support as a key mechanism for promoting integrated mental health care for refugees, as reflected in themes from the REACH project that emphasise the value of community support	This happens when clients may not immediately trust mental health care providers and/or are new to the mental health care system, leading to hesitancy and caution during the treatment process	Recognising that promoting trust and connection through culturally resonant, community-based, co-produced strategic approaches is often more effective than rigid adherence to traditional, individual-focused mental health care models highlights the importance of alternative models of care. These approaches place greater emphasis on the social determinants of health—such as social support—acknowledging	For refugee communities, mental health support is more accessible and less stigmatised when embedded in familiar, communal contexts rather than framed through clinical language. Building trust through informal, culturally attuned engagement and co-produced strategies lays the groundwork for deeper therapeutic relationships and more sustained care participation.

	support may scare people away. "	and community-driven interventions.		their critical role in fostering mental wellbeing among refugee populations.	
Participants shared how mental health becomes more acceptable and accessible when integrated into everyday community support, reducing stigma.	*"Yeah, I have one last comment like I think sometimes it's also a good idea to like you offer program with intention of like mental health support, but they should not be like highlighted, you know like find a way that you can still offer the support. It could be like maybe getting together at a park, an example would be like that would still bring the communities together, they could speak their language, things like that. Because sometimes, like just naming it mental health support may scare people away. So you kind of have to find how to bring them together and also keep them in that journey if they need that. So I think if you could do that, that would be also good."*	It is plausible to claim that community-based mental health approaches align more closely with refugees' pre-existing cultural expectations of care, thereby increasing their engagement and willingness to use services.	The inferred phenomenon is dependent upon the presence of cultural norms that emphasise collective support and social connection, where individuals feel more comfortable and engaged in care when it is provided in a familiar, community-oriented setting rather than through Western individualistic models.	See above	See above

**Community Health Center**								
AAB				MH				*Agreement*
Exp	*Quote*	Inf	Dis	Exp	*Quote*	Inf	Dis	**Final**
1.4. Participants in this dialogue highlighted the effects of overwhelmed services, burnout, and limited resources	*"And I think the main one of the main challenges is because, well, at least what I'm noticing, there's a lot of burnout in our wider profession. You know, there's new people that you*	1.4 It is plausible to claim that =<br>Lack of resources staff can lead to increased pressure on the primary health care system ultimately leading to lack of mental health resources for refugee clients.	1.4 The inferred phenomenon is dependent upon the presence of a resource-strained and high-stress healthcare environment,	Participant describes how burnout is pervasive in the system, and that the constant need to develop relationships with rotating and	*"there's a lot of burnout in our wider profession. You know, there's new people that you have to rebuild their relationship with all the time and different service providers, and it takes time to*	Relationship is a mechanism for integrated health and social care services but is obfuscated by lean funding	This is dependent on the industry's ability to retain personnel, and capacity to train and mobilise newer providers such that the rate at which they replenish workforce loss is slower than the rate of loss	*High levels of burnout, staff turnover, and limited resources in the care system contributes to disengagement for providers and users, fuelled by shifting staff and fractured ability to create consistency and trusting relationships with clients.*

leading to disengagement from both providers and refugees.	*have to rebuild their relationship with all the time and different service providers, and it takes time to and programs also change."*		where chronic understaffing and systemic instability generate frustration, helplessness, and disengagement among all stakeholders.	overworked staff creates internal challenges	*and programs also change. So that's one of the challenges that we see"*	and lack of funding for delivering comprehensive services		**Resource-strained, high stress working environments makes it difficult to retain personnel. Rapidly changing workforces makes it more difficult for clients to build rapport with the staff and thus create trusting and productive working relationships with their providers.**
Participants in this dialogue mentioned that when care is easily accessible, it promotes better relationships, trust, and improved mental health outcomes.	*"Because they like a very low barrier in terms of who they accept and have people who speak the same language and specify on people with like lived experiences of violence through migration."*	It is plausible to claim that increasing resources? reducing barriers to healthcare strengthens client-provider relationships, enhances trust, and creates a system where individuals feel understood and supported in a culturally responsive way.	The inferred phenomenon is dependent upon the fundamental human need for social connection, where individuals seek and engage more effectively in care when they feel represented, understood, and integrated within a system that acknowledges their cultural and personal experiences.	Participants discuss the importance of similar language, shared lived experiences and the role of culture in overcoming service barriers: that having these resources readily available offers a rare pathway into care for linguistic and cultural minorities	*"Often it's actually just [REFUGEE ADVOCACY ORGANIZATION] and I think having more services like [REFUGEE ADVOCACY ORGANIZATION] would be really great like where they have counsellors who speak the same language of folks and they specify in refugee experiences. Just like more of [REFUGEE ADVOCACY ORGANIZATION] *laughs* would be great because they like a very low barrier in terms of who they accept and have people who speak the same language and specify on people with like lived experiences of violence through migration."*	Care for refugees with linguistic and cultural l needs is enabled by providers who share similar language backgrounds to the client, which can lead to a stronger patient/provider relationship	This happens when clients may exercise caution or harbour mistrust for the mental health care system due to lack of familiarity, stigma, and/or perception of the provider's ability to understand their needs	*Access and familiarity can help foster trust and improve the provider/patient relationship, and can be enabled by shared language, culture, and understanding.*<br><br>**When care is linguistically and culturally accessible, trust is fostered between patients and providers, leading to stronger and more effective patient engagement and satisfaction.**
Participants in this dialogue raised the issue around alienation and disengagement	*"They came from their homelands, you know, from a very different world views. Then all of a sudden they're here in a very western,*	It is plausible to claim that Western, individualistic approaches to mental health care may not align with the health care expectations and cultural	The inferred phenomenon is dependent upon the existence of influence of cultural norms	Participants discuss the importance of language and the difference between interpretation and	*"Some cultures don't even have the word for feeling. You know I worked with a Rohingya for a while. When you ask somebody from the Rohingya*	Different cultural groups express emotional pain or mental health challenges	This is dependent on the patient coming from a different culture of care and social norms, leading to a stark difference in expressiveness that	*Mental health services that over rely on Western, individualistic frameworks may fail to resonate with refugees who may come from community-focused care.*

caused when mental health services impose Western frameworks on refugees who come from community-based traditions.	*individualistic worldviews even though they're with other refugee folks from different parts of the world. But now they feel incredibly isolated, facing multiple socioeconomic barriers, discrimination, etc., and they're isolated." Speaker*	understandings of refugees, leading to disengagement and mistrust of services or delayed care.	and differing perceptions of mental health, where discussing mental health openly may be culturally inappropriate or misunderstood, creating a barrier to engagement with care.	translation. Participant notes that due to a difference in cultural understanding, there is a miscommunication which leads to an inappropriate referral	*community 'how do you feel?' They'll say 'my head hurts', right? "*	differently; lack of understanding can lead to barriers in access for mental health or appropriate interventions	contrasts with the host country's way of understanding of emotion	**Mental health services that rely on Western, individualistic models of care may alienate refugees whose care expectations are rooted in community, leading to miscommunication and possibly lack of engagement from services altogether.**
Participants in this dialogue endorsed the role of community-based, non-Western approaches in facilitating disclosure, trust, and improved mental health outcomes.	*"Sometimes the antidote is community connection in a group setting as opposed to an individual counselling setting. But I think, you know, having said that they're desperate enough that a lot of people take what they can get to get help. But culturally it is pretty western in point of view."*	It is plausible to claim that community-based mental health approaches align more closely with refugees' pre-existing cultural expectations of care, thereby increasing trust and willingness to use services.	The inferred phenomenon is dependent upon the presence of cultural norms that emphasise collective support and social connection, where individuals feel more comfortable and trust in care when it is provided in a familiar, community-oriented setting rather than through Western individualistic models e.g., one on one counselling.	Participant explains the challenges that refugees face in a new health care system and new country, and how programmes that build social connection helps create community connection which is more of	*"They came from their homelands, you know, from a very different world views. Then all of a sudden they're here in a very western, individualistic worldviews even though they're with other refugee folks from different parts of the world. But now they feel incredibly isolated, facing multiple socioeconomic barriers, discrimination, etc., and they're isolated. So programs like maybe Rainbow Refugee or the programs that we have here that really rebuild that social connection while also reconnecting all of us with each other have been really helpful for a lot of the folks that I serve"*	Refugees who require more community-centric forms of care are better served when programmes help refugees foster relationships with other clients who share lived experiences	This happens when who come from forced migration backgrounds are isolated resources such as community-based interventions where clients shared lived experience increase social support	
