# Supplementary material for: Integrated mental health for refugees: A realist theory building study
Source: PLOS Ment Health. 2026 Jan 30;3(1):e0000547. doi: 10.1371/journal.pmen.0000547 (PMC12857968; doi:10.1371/journal.pmen.0000547)
Supplement: Attachment — Submitted filename: response to reviewers.pdf [file pmen.0000547.s005.pdf]

School of Nursing | Faculty of Health | University of Victoria
HSD Building A402a PO Box 1700 STN CSC Victoria BC V8W 2Y2 Canada
Phone: 250-721-7954 | Fax: 250-721-6231 | uvic.ca/health/nursing

To: Jenna Scaramanga
Staff Editor
PLOS Mental Health

Re: PMEN-D-25-00314
Integrated Mental Health for Refugees: A Realist Theory Building Study
PLOS Mental Health

Dear editorial reviewers, thank you for providing feedback on how to improve our manuscript and guidance on addressing the points raised during the review process. We believe that the recommendations have strengthened our manuscript. Our response to the review is addressed in this letter and in our updated manuscript. Responses to the reviewers are *italicized*.

The reviewer notes that our manuscript partially fulfils PLOS Mental Health's publication criteria. We believe that changes to the manuscript have strengthened the presentation of our research and that it meets standards for research integrity. Specifically, our manuscript contributes to research **gaps in theorization of integrated mental health care**.

The reviewer notes that we have not made all data available as part of the supporting information, however we have provided our analysis documents in supplementary files including how we developed themes for the program theory, see S1 supplement (SI Synergy table) we have also added (S2 figure consensus building exercise). Although the original transcripts are coded, they are not provided because they may contain confidential material about research participants.

To address the reviewer comments related to "superficial coverage of the relevant literature". We have now included a description of refugees and asylum seekers. Please note however that these groups are not homogenous and that not all refugees and asylum seekers develop mental health conditions, rather risk factors are associated with insufficient and tailored mental health support.

Our manuscript seeks to investigate and draw attention to the need for responsive healthcare systems; thus, the review is to provide an overview of what we know about what works in terms of integration of health services and systems to improve health outcomes, specifically mental health access and equitable care for refugees. To this end we have included extra literature in the introduction section, and the methods sections totaling 24 new citations.

Reviewer #1: Thank you for the opportunity to review "Integrated Mental Health for Refugees: A Realist Theory Building Study" for possible publication in PLOS Mental Health. This is a relevant paper that explores the development of an initial program theory on the integration of refugee mental health across services. Research on this topic holds the potential to improve our understanding of how-to better support people with forced migration backgrounds. However, some recommendations are offered to be addressed before this paper can be accepted for publication. I hope the comments are useful to you in improving the manuscript for publication.

School of Nursing | Faculty of Health | University of Victoria
HSD Building A402a PO Box 1700 STN CSC Victoria BC V8W 2Y2 Canada
Phone: 250-721-7954 | Fax: 250-721-6231 | uvic.ca/health/nursing

First, the manuscript provides a rather superficial coverage of the relevant literature. The section could be improved by providing an overall description of the refugee and asylum seekers characteristics.

We provide a definition of refugee and asylum seekers in the introduction. We also include a section of relevant literature to background and literature view under section 'known mechanisms related to integrated care'. We felt it was first important to foreground realist methodology by describing conceptual foundations of a realist approach. The literature review starts from lines 119-217.

The introduction section could also benefit from specific examples regarding some of the information presented. For example, a detailed description of cultural stigma along with examples.

Thank you for this insight, we agree that cultural stigma while important detracts form the systems perspective we are trying to advance, i.e. responsiveness of the broader healthcare system. To focus on only cultural stigma may reinforce individualistic perspectives and micro level (individual) context; without examining the nuanced and intersections of broader cultural practice. To address this, we have included studies which argue that we need to instead look at meso (organizational) and macro (systemic) contexts and responsiveness of healthcare systems toward promoting refugee health. Moreover, there is a danger in highlighting cultural stigma because it may abdicate responsibility of policy actors, we have briefly discussed cultural stigma as being highly contextual, and related to other factors that are political, social and broader cultural processes. Cultural stigma is addressed in lines, 64-67; 102;774-778 and 902.

Similarly, the authors should describe interventions that have shown to work with this population.

*Thank you, we have described interventions that work under the section '**Known mechanisms of integrated care'**, this includes added references**.** See lines 171-271. We are interested to understand why interventions work, in what context and why? Rather than what works. Interventions may not work across all contexts because integrated care mechanisms maybe context specific. To this end we have strived to co develop an initial program theory by adopting a realist methodology about what works across complex health care systems, how they function to promote integration of care.*

For a better reading, please carefully review the organization of the introduction section avoiding unnecessary repetitiveness to improve its flow.

Noted. We have edited out repetitive sections. We hope this improves the flow.

School of Nursing | Faculty of Health | University of Victoria
HSD Building A402a PO Box 1700 STN CSC Victoria BC V8W 2Y2 Canada
Phone: 250-721-7954 | Fax: 250-721-6231 | uvic.ca/health/nursing

The authors state this study implemented a participatory research approach but provide very detail about it. If this study was designed as such, the procedure should be described in detail.

Thank you for alerting us to this lack of description. We have provided more information about our approach see: section ***'Study design and setting'****. Here we explain how we apply principles of community based participatory research (CBPR) in our study design and work with community. Participatory approaches in realist approaches have not been well defined however we were reflexive of the need to highlight interest group holder involvement and contribution to ongoing theory development and included additional articles to support our choices. See lines 252-292 and added description on Participant recruitment and description.*

More description of the recruitment process and justification of such is necessary. A strong justification for the sample size is missing. The eligibility criteria are not explicitly stated, please provide this information and justification for such.

*Thank you, we have provided a description of our purposive sampling strategy under section '****Participant recruitment and description'****. See lines 293-309.*

Also, the authors do not provide sufficient description of the characteristics of the participants.

The participants were purposively sampled, and we were not aiming to collect their socio demographic information, however we did provide a table that described were participants were situated and which agencies they represented and the kind of work they were engaged in, e.g. settlement, primary health care and so on. We did not collect specific criteria as to year of employment or professional backgrounds beyond their role within the organization. This may be a limitation of the study design and is addressed as such under limitations.

Importantly, the format of the dialogue sessions is very vaguely described, leaving the reader with a very unclear picture of how these developed.

Thank you we describe the dialogue sessions occurring online virtually through Zoom meeting and or in person. We also describe that participants with lived experience were interviewed together and apart from service providers. Although many of these roles are ambiguous because most refugee participants in this research were also providing health service navigation support to peers. Dialogue sessions were semi structured interviews either individually or as a focus group. Described under methods.

I appreciate the authors detailed description and justification of their qualitative analysis. However, they do not provide the authors' positionality which would enrich the paper, for example describing how their positionalities impacted the group experience and research overall following suggestions outlined by Grzanka & Moradi (2021) re: positionality statements:

Grzanka, P. R., & Moradi, B. (2021). The qualitative imagination in counseling psychology: Enhancing methodological rigor across methods. Journal of Counseling Psychology, 68(3), 247

*Thank you for this recommendation, this was a very interesting article on values behind the methods in psychological research. The section on researcher reflexivity and positionality are poignant. We have now included a brief description of researcher positionality under the section **'Study design and setting'**. This is part of our principles of CBPR. However, it should be noted that most realist methods do not explicitly draw attention to researcher positionality, so this is novel and necessary when engaging in participatory research methods. We have also included other references to augment this section. We have however tried to explain our positionality as per Grzanka & Moradi (2021) in terms of how we approached our analysis in relation to our positionality and experience using a realist method. Of course, no research is value neutral and that is why we also applied Wiltshire and Ronkainen's approach to realist and thematic analysis together (realist providing the ontological depth and thematic making sense of regular patterns in the data). See lines 262-268 re researcher positionality statements.*

The authors provide quotes from participants, but I suggest adding information about the participant such as their gender and age.

Gender and age were not variables of interest to the outcomes of a theory building research; rather we were focused on mechanisms that promote system change. We did not collect this kind of data and do not see this as a study limitation due to the realist methodology used to co develop an initial program theory. We did not employ a gender based on intersectional analysis to this study because we simply wanted to glean perspectives of the diverse participants from respective agencies and services. This issue seems to be more about power relations and reflects researcher positionality and is addressed in limitations section.

Some of the qualitative results are very repetitive, the authors could instead use the space to expand of their presentation of the findings.

Yes, in some cases our findings overlap as is typical when presenting thematic analysis there can be many overlapping themes. Findings are presented in terms of initial program theory development. We wanted to show how we applied realist reasoning to get to underlying causes i.e. key mechanisms thought to cause change. To provide clarity we present the findings in our IPT model which helps differentiate mechanisms of action across client, provider and service levels see figure no.3. We have added headings and edited sections in the discission section.

There seems to be a lot of overlap in the results and discussion sections, the discussion section reads a bit more like a recapitulation of the results section. Please revise to be less descriptive of results and more synthesized with the extant literature in line with this goal.

School of Nursing | Faculty of Health | University of Victoria
HSD Building A402a PO Box 1700 STN CSC Victoria BC V8W 2Y2 Canada
Phone: 250-721-7954 | Fax: 250-721-6231 | uvic.ca/health/nursing

This recommendation is the same as the mention that our findings are repetitive. We have revised the findings section however we engaged in theoretical validity where we analyzed our data in relation to existing research on realist approaches to uncover known mechanisms of action to promote integrated care; not all are related to mental health as the concept of integrated care may be broader and yet have relevance specifically for integrated mental health care for forced migrant population groups. To this end we aimed to synthesize the literature to advance realist understanding of theories that could inform the deeper layers of what triggers actors to behave in positive ways to promote integrated care; for example, we hypothesized intersectionality and health equity theory as potentially contributing to positive behaviors such as advocacy moral commitment. To streamline we have included another section on 'Candidate Theory' in the discussion section to break up the repetitiveness. See lines 838-871.

The authors do a good job articulating the limitations present in the study.

Thankyou,

This concludes our response to the review. We hope you find these major revisions acceptable for potential publication with PLOS Mental Health. We believe it will contribute to research in migrant mental health and understanding the mechanisms that promote better integrated care and services for refugees.

Thank you for the opportunity to resubmit our manuscript and we look forward in hearing from you,

Kind regards,

Nancy Clark

Nancy Clark PhD RN
