# Supplementary material for: Integrated mental health for refugees: A realist theory building study
Source: PLOS Ment Health. 2026 Jan 30;3(1):e0000547. doi: 10.1371/journal.pmen.0000547 (PMC12857968; doi:10.1371/journal.pmen.0000547)
Supplement: Attachment — Submitted filename: Letter response to Reviewer (1).pdf [file pmen.0000547.s006.pdf]

School of Nursing | Faculty of Health | University of Victoria
HSD Building A402a PO Box 1700 STN CSC Victoria BC V8W 2Y2 Canada
Phone: 250-721-7954 | Fax: 250-721-6231 | uvic.ca/health/nursing

December 26, 2025

Jason Cameron McIntyre, PhD; B App Sc; B Psy Sc (Hons I)
Academic Editor
PLOS Mental Health

Dear Editors:

Re: PMEN-D-25-00314 Integrated Mental Health for Refugees: A Realist Theory Building Study

Dear editorial reviewers, thank you again for your detailed comments and recommendations which has strenthened our manuscript. I have reviewed and made thorough edits based on reviewer one's feedback. Our response to the review is addressed in this letter and in our updated manuscript. Responses to the reviewers are *italicized*.

Comment: However, the added sentence now makes the following section somewhat repetitive: "The lack of integration of care across systems and services can lead to increased healthcare costs and poor mental health for forced migrants [14]. These challenges can result in inequitable access to and cost of mental health care and poor mental health outcomes [6]."

Response: We have removed the second sentence to avoid repetition.The removal and edits to this sentence can be found in lines 74-79 in marked copy of the manuscript which highlights the track changes.

Comment: "the positionality of the second author "AAB (second author) is …" is currently missing and should be included for completeness and consistency."

Response: We have added the positionality of AAB (second author)- it seems that we had done this edit in the previous submission but it did not come through. Please find the edits in lines 255-257 in marked copy of the manuscript which highlights the track changes.

Comment: "Importantly, the format of the dialogue sessions remains very vaguely described. The authors state that "dialogue sessions were semi structured interviews" but it would be important to know what kinds of questions were included in such interviews."

Response: Thank you for point this out. We have described and defined the deliberative dialogue sessions and interview questions. Please see lines 308-310, and 321-323. We have also uploaded a supplement material with the script for the interview transcript with questions (see S1 Text.Interview Guide). In marked copy of the manuscript which highlights the track changes.

Comment: "The Analysis section is thorough, but it could be more succinct to ensure the manuscript remains within the journal's length specifications. Additionally, the statement, "We

School of Nursing | Faculty of Health | University of Victoria
HSD Building A402a PO Box 1700 STN CSC Victoria BC V8W 2Y2 Canada
Phone: 250-721-7954 | Fax: 250-721-6231 | uvic.ca/health/nursing

applied a realist phraseology to create explanatory statements [71],” would benefit from brief clarification to support reader understanding, particularly for those less familiar with realist methodology.”

Response: We have edited repetivness in the analysis section, see 403-408. in marked copy of the manuscript which highlights the track changes. We have also clarified what is meant by realist phraseology, please see lines 440-446 in marked copy of the manuscript which highlights the track changes.

Comment: “Finally, in the presentation of themes, the “M” (mechanism) component appears to be missing for several themes, specifically stagnation, connection, and burnout. Addressing this will improve clarity and consistency.”

Response: *Thanks again for pointing this out. We have added “M” (mechanism) for themes of stagnation, connection and burnout. We have also re described the themes as micro theories fitting with realist philosophy, i.e., initial program theory development.*

Thank you again for all of the insights and recommedations. You will note that we have made further edits for repitition throughout the paper with the aims of making it more succinct.We have reviewed and edited the reference accordingly but have not added new references.

Please also note that we have submitted the 3 figures into the PLOS’s NAAS tool and they have been converted to TIFF file type.

We believe we have addressed all of the reviewer comments, recommendations and edits which have greatly strengthened this manuscript.

Please let us know if you have further questions,

Sincereley,

Nancy Clark

Nancy Clark RN PhD
